# Microbial community response to hydrocarbon exposure in iron oxide mats: an environmental study

**DOI:** 10.3389/fmicb.2024.1388973

**Published:** 2024-05-10

**Authors:** Chequita N. Brooks, Erin K. Field

**Affiliations:** ^1^Department of Biology, East Carolina University, Greenville, NC, United States; ^2^Louisiana Universities Marine Consortium, Chauvin, LA, United States

**Keywords:** iron mat, iron-oxidizing bacteria, hydrocarbons, microbial community, biogeochemistry

## Abstract

Hydrocarbon pollution is a widespread issue in both groundwater and surface-water systems; however, research on remediation at the interface of these two systems is limited. This interface is the oxic–anoxic boundary, where hydrocarbon pollutant from contaminated groundwaters flows into surface waters and iron mats are formed by microaerophilic iron-oxidizing bacteria. Iron mats are highly chemically adsorptive and host a diverse community of microbes. To elucidate the effect of hydrocarbon exposure on iron mat geochemistry and microbial community structure and function, we sampled iron mats both upstream and downstream from a leaking underground storage tank. Hydrocarbon-exposed iron mats had significantly higher concentrations of oxidized iron and significantly lower dissolved organic carbon and total dissolved phosphate than unexposed iron mats. A strong negative correlation between dissolved phosphate and benzene was observed in the hydrocarbon-exposed iron mats and water samples. There were positive correlations between iron and other hydrocarbons with benzene in the hydrocarbon-exposed iron mats, which was unique from water samples. The hydrocarbon-exposed iron mats represented two types, flocculent and seep, which had significantly different concentrations of iron, hydrocarbons, and phosphate, indicating that iron mat is also an important context in studies of freshwater mats. Using constrained ordination, we found the best predictors for community structure to be dissolved oxygen, pH, and benzene. Alpha diversity and evenness were significantly lower in hydrocarbon-exposed iron mats than unexposed mats. Using 16S rDNA amplicon sequences, we found evidence of three putative nitrate-reducing iron-oxidizing taxa in microaerophile-dominated iron mats (*Azospira, Paracoccus*, and *Thermomonas*). 16S rDNA amplicons also indicated the presence of taxa that are associated with hydrocarbon degradation. Benzene remediation-associated genes were found using metagenomic analysis both in exposed and unexposed iron mats. Furthermore, the results indicated that season (summer vs. spring) exacerbates the negative effect of hydrocarbon exposure on community diversity and evenness and led to the increased abundance of numerous OTUs. This study represents the first of its kind to attempt to understand how contaminant exposure, specifically hydrocarbons, influences the geochemistry and microbial community of freshwater iron mats and further develops our understanding of hydrocarbon remediation at the land–water interface.

## Introduction

Hydrocarbon pollution is an international issue from the Deepwater Horizon Oil Spill to 500,000+ underground storage tanks leaking oil into groundwaters (as of September 2020) (Meegoda and Hu, 2011; Office of Underground Storage Tanks, D.C., 2020). However, an effective and efficient clean-up method for these contaminants has yet to be developed. This is highly problematic, as groundwaters can release toxic hydrocarbons into public drinking water or aboveground recreational waterways. In turn, hydrocarbons, such as benzene, can be highly hazardous to human health, resulting in leukemia and anemia (Badham and Winn, 2007). To combat this long-standing public health crisis, there is a plethora of work focused on hydrocarbon biodegradation. Efforts have usually focused on benzene, as it is highly mobile in groundwater (Minetti et al., 2017) and resistant to oxidation and degradation (Johnson et al., 2003). Benzene biodegradation more readily occurs under aerobic conditions, and it occurs via well-studied pathways (Jindrová et al., 2002). However, the application of aerobic degradation pathways is limited as oxygen is quickly expended in the water column leading to anoxia in contaminated zones (Keller et al., 2018). Because it is not limited by oxygen dissolution in groundwater, anaerobic benzene degradation has also been a focus of research (Keller et al., 2018). Previous terrestrial studies have focused primarily on groundwater environments, but approaches that attempt to incorporate the oxic–anoxic boundary are notably limited (Bradley, 2012). In a study using beach sand from Pensacola Beach, FL, an oxic–anoxic incubation model was found to increase the efficacy of the aerobic hydrocarbon degradation, as aerobic bioremediation was bolstered by the byproducts of anaerobic metabolisms produced during anoxic periods (Karthikeyan et al., 2020). As “oxic” and “anoxic” conditions are concurrent in the iron mat, it is possible that similar bolstering of hydrocarbon degradation occurs over time within this microbial community. Furthermore, the oxic–anoxic boundary where groundwater meets surface water is the last stop prior to widespread contamination by hydrocarbons. In this way, iron mats could be considered the last chance for hydrocarbon remediation using microbial communities before it outspreads.

The use of microorganisms to degrade hydrocarbons does not come without other challenges. Hydrocarbons increase stress in the environment by leading to the production of reactive oxygen species, which can damage microbial DNA (Liu et al., 2019). This, in turn, can impact the structure of microbial communities exposed to hydrocarbons by decreasing community diversity (Maila et al., 2005; Máthé et al., 2012; Sun et al., 2013). In a study of cyanobacterial mats in Berre lagoon, France, hydrocarbon exposure decreased the influence of seasonality (Aubé et al., 2016) possibly due to the tendency for hydrocarbon-inundated communities to skew toward more extremophilic organisms (Paissé et al., 2008; Abed et al., 2014). Microbial communities exposed to hydrocarbon contamination can also decrease in alpha diversity as a result of the decreased probability of horizontal gene transfer compared with communities exposed to other contaminants, such as heavy metals or antibiotics (Máthé et al., 2012).

The microbial communities in freshwater iron mats are already exposed to high concentrations of heavy metals and increased environmental stress, since iron-oxyhydroxides (FeOOH) produced by iron-oxidizing bacteria (FeOB) adsorb heavy metals (Field et al., 2019; Li et al., 2020), aromatic carbons (Baskar et al., 2012), phosphorous (Takeda et al., 2010; Buliauskaitė et al., 2020), and hydrophilic pesticides (Søgaard et al., 2001) from the water column. These environmental contaminants can easily desorb from the FeOOH when environmental conditions lead to changes in pH, ionic strength, oxygen concentrations, or flow (Little et al., 2016), which may lead to acute stress in iron mat communities. Due to the adsorptive properties of biogenic FeOOH, iron mats have been suggested to be useful in removing benzene and other hydrocarbons from contaminated sites (Abbas et al., 2016; Brooks and Field, 2020). The chemical properties of iron mats are not the only source of potential for benzene removal. There have been multiple studies that show involvement in the biodegradation of benzene and other hydrocarbons from functional groups such as sulfate-reducing bacteria (SRB) (Edwards and Grbić-Galić, 1992; Chakraborty and Coates, 2004; Keller et al., 2018), iron-reducing bacteria (FeRB) (Lovley et al., 1996; Jahn et al., 2005; Tremblay and Zhang, 2020), and nitrate-reducing bacteria (NRB) (Coates et al., 2001; Fahy et al., 2008; Atashgahi et al., 2018), which have been previously identified in iron mats. If hydrocarbon-degrading organisms are present and active, the iron mat community could prove to be an invaluable resource in application of hydrocarbon remediation at the oxic–anoxic boundary. Here, we present study from *in situ* sampling of iron mats that have been chronically exposed to benzene contamination in Town Creek, Greenville, NC.

In this environmental study, we have paired geochemical and molecular data to establish a baseline understanding of how hydrocarbons impact iron mats. We present how the presence of hydrocarbons correlates with geochemical condition, microbial community structure, and microbial functional potential in iron mats. This study serves as the beginning of our understanding of how FeOB-driven communities respond to hydrocarbon exposure, which may lead to future advancement in hydrocarbon remediation at the oxic–anoxic interface.

## Materials and methods

### Site description, sample collection, and geochemical analyses

**Figure 1:**
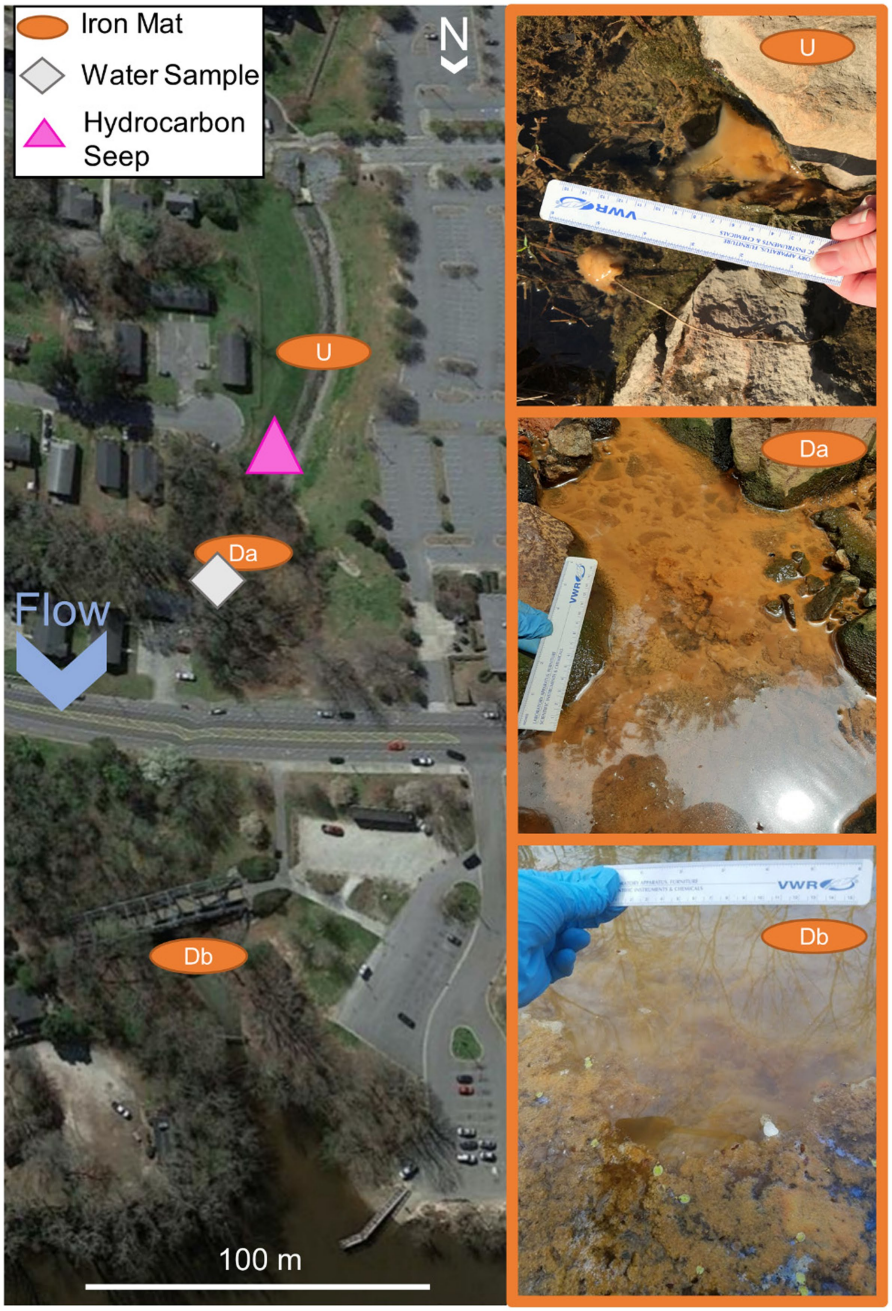
A map of Town Creek, Greenville, NC. Iron mat sampling locations indicated with orange ovals labeled with iron mat location ID: Upstream (U) unexposed, Downstream A (Da) hydrocarbon-exposed, or Downstream B (Db) hydrocarbon-exposed. The water sampling site (W) hydrocarbon-exposed is indicated by a gray diamond and was chosen so as to be as far across the creek cross-section from iron mat sample as possible to avoid confounding results. The hydrocarbon seeps are indicated by the pink triangle. Seep location based on the study by Humphrey et al. (2018). Map obtained from Google Earth Pro v. 7.3.3.7786 and modified with iron mat, benzene seep, and water sample locations and inset images.

The creek site, Town Creek, Greenville, NC (Figure 1), is in a residential area and consists of a low-flow creek with high banks lined with riprap. Sampling of iron mats and water samples took place over four time points for 2 years in March of 2018 and July and August of 2019. Samples were collected from upstream, unexposed iron mats (U) and downstream, hydrocarbon-exposed iron mats (Da, Db). Samples for biological molecular analysis were collected aseptically and stored on ice until they were transported to the laboratory and stored at −80°C. Samples of iron mats were also processed via filtration in an acid-washed top-bottle filter using pre-ashed (500°C, 4 h) Whatman glass microfiber filters, Grade 934-AH (1.5 μm pore size, GE Healthcare Bio-Sciences, Marlborough, MA), and filtrates were stored on ice and transported to the laboratory where they were stored at −20°C until they could be analyzed. Sample analysis for phosphate, ammonia, nitrates/nitrites (SmartChem 170 and 200 Discrete Analyzer, Unity Scientific), and dissolved organic carbon (DOC) (TOC-LCPH/CPN PC-Controlled TOC Analyzer and ASI-L Autosampler, Shimadzu Scientific Instruments, Inc.) was carried out by the Environmental Research Laboratory at East Carolina University, Greenville, NC. Iron mat was also collected in pre-treated bottles containing ascorbic acid provided by the Environment 1, Inc. lab in Greenville, NC. Immediately after collection, hydrochloric acid was added to the samples, and they were stored at 4°C until they were analyzed using the EPA method 602 (Warner et al., 1984). Measurements of total iron, oxidized iron (Fe^3+^), and reduced iron (Fe^2+^) for all sampling sites were conducted immediately followed by sampling using the ferrozine method (Lovley and Phillips, 1987). Measurements of pH, conductivity, dissolved oxygen, and water temperature were taken using a YSI Quatro Professional Plus (YSI Inc., Yellow Springs, OH).

### DNA extraction, 16S rDNA sequencing, and phylogenetic analysis

The QIAGEN DNeasy PowerSoil Kit (Qiagen, Germantown, MD) was used according to the manufacturer’s instructions for each mat sample with the following modifications: DNA was eluted in 60 μL and cell lysis occurred using a 10-min cycle in a Disruptor Genie (Scientific Industries, Inc., Bohemia, NY) set to maximum speed. 16S rDNA sequencing of the V4-V5 region was performed at the Comparative Genomics and Evolutionary Bioinformatics’ Integrated Microbiome Resource (CGEB-IMR, Halifax, NS) using universal primers 515FB and 926R (Parada et al., 2016; Walters et al., 2016). Sequences were processed and annotated using mothur v. 1.44.1 (Schloss et al., 2009, 2011; Kozich et al., 2013) and the SILVA database v. 138.1 (Quast et al., 2013). The MiSeq SOP was accessed on 13 April 2020^1^ and used to identify present taxa (97% OTU threshold). Further analyses were performed in R v. 3.5.2 using phyloseq v. 1.26.1 (McMurdie and Holmes, 2013) to import mothur data into R, perform quality checks, calculate alpha diversity indices, ordination, and calculate relative abundances. All samples were rarified to even depth (sample size of 6,883) based on the smallest sample with a set seed prior to alpha diversity and evenness calculations. The package microbiome v. 1.4.2 (Lahti et al., 2017) was used to convert data into a centered log ratio format for beta diversity indices and calculate Pielou’s Evenness. Before calculating beta diversity indices, a centered log ratio was used to transform the count data into dominance of taxa compared with the geometric mean of all taxa on a log scale, and the principle coordinates analysis was performed using a redundancy analysis (RDA). The package vegan v. 2.5–6 (Oksanen et al., 2019) was used to convert phyloseq objects into Euclidean distance and beta dispersion and calculate statistics for the Canonical Correspondence Analysis (CCA). A permutation ANOVA (PERMANOVA) was used to test each of the margins (factors) (number of permutations = 9,999) for variance inflation factors, calculated using *vif.cca()*. CCA was chosen to show overall community structuring as it was most useful in building a model of community structuring in response to the numerous environmental factors measured. The package picante v. 1.8.2 (Kembel et al., 2010) was used to create a data frame with alpha diversity measurements. The package edgeR v. 3.24.3 (Robinson et al., 2009; McCarthy et al., 2012) was used to calculate the log-fold change (logFC) of OTUs between benzene exposed and unexposed sites. Phyloseq objects were converted to edgeR objects using the phyloseq extension accessed on 6 January 2020.^2^ Samples were filtered for independence using a variance threshold set to 1e^−7^ prior to calculating logFC. Visuals were generated using ggplot2 v. 3.3.2 (Wickham, 2016), and relative abundances were converted to percentages for visualization using the package scales v. 1.1.1 (Wickham and Seidel, 2020). Color blind accessible palettes were applied to graphs using ggthemes v. 4.2.0 (Ichihara et al., 2008; Chang, 2013; Arnold, 2019).

### Metagenomic sequencing, assembly, and metagenomic-assembled genome annotation

DNA extraction methods for metagenomic sequencing were performed for 16S rDNA amplicon sequencing. Metagenomes were sequenced at CGEB-IMR using the Illumina Nextera Flex for the NextSeq at 3x depth paired-end reads. Sequence adapters were trimmed using TrimGalore v. 0.4.5 (Krueger, 2015), and sequence loss was assessed using FastQC v. 0.11.5 (Andrews, 2010). Reads were assembled using SPAdes v. 3.13.0 (Bankevich et al., 2012), and unpaired reads were preserved. Assembly quality was assessed using the MetaQUAST function in QUAST v. 5.0.2 (Gurevich et al., 2013) (Supplementary Table S5). Assemblies were binned into Metagenomic Assembled Genomes (MAGs) using MaxBin v. 2.2.7 (Wu et al., 2014), CONCOCT v. 1.0.0 (Alneberg et al., 2013), and Metabat2 v. 2.14 (Kang et al., 2019). All bins were aggregated using DAS Tool v. 1.1.2 (Sieber, 2017). The quality of each MAG was checked using the CheckM v. 1.0.18 (Parks et al., 2015) lineage-specific workflow (Supplementary File S3). Bin identities of MAGs (> 59% complete, < 10% contamination) were determined using MetaSanity v. 1.2.0 (Neely et al., 2020) PhyloSanity pipeline. MAG genome size and GC content were calculated using the RASTtk server (Aziz et al., 2008; Overbeek et al., 2014; Brettin et al., 2015) accessed on January 2021.

Assembled MAG FASTA files were imported into the online user interface for KBase (Arkin et al., 2018) on 28 December 2021. MAG classifications were verified using “Classify Microbes with GTDB-Tk – v.1.7.0” on 29 December 2021 (Chaumeil et al., 2020). MAG assemblies were annotated using “Annotate and Distill Assemblies with DRAM” on 19 May 2022 (Shaffer et al., 2020). Representative FeOB isolate genomes were sourced from NCBI on 21 February 2022. Assemblies were grouped in KBase using “Build AssemblySet – v1.0.1” and annotated with “Annotate and Distill Assemblies with DRAM” on 16 May 2022. Figures were compiled and edited in Vega-Lite (Satyanarayan et al., 2017). Representative FeOB assemblies chosen were BioProject: PRJNA32827 (*Gallionella capsiferriformans* ES-2 (b-proteobacteria)) (D. Emerson, unpublished), BioProject: PRJNA13615 (*Mariprofundus ferrooxydans* PV-1) (Emerson and Moyer Craig, 2002), BioProject: PRJNA56115 (*Leptothrix ochracea* L12) (E. Fleming, unpublished), BioProject: PRJNA224116 (*Ferriphaselus amnicola* strain OYT1 chromosome, complete genome) (S. Kato, unpublished), and BioProject: PRJNA542651 [*Mariprofundus erugo* (proteobacteria)] (Garrison et al., 2019).

### Hidden Markov models

Assembled and unpaired reads from SPAdes were filtered to remove contigs with fewer than 500 base pairs using filter_contigs.py (accessed 2020 AUG 5; https://github.com/tinybio/filter_contigs) and was subsequently filtered for contigs that had at least 1.5x coverage using another python script (accessed 2020 AUG 5; https://microsizedmind.wordpress.com/2015/03/05/removing-small-low-coverage-contigs-from-a-spades-assembly/) (Gihawi et al., 2019). The remaining contigs were annotated by prokka v. 1.14.5 (Seemann, 2014) using the *--metagenome* flag. The UniProtKB database (accessed 2021 JAN 28; https://www.uniprot.org/) (Boutet et al., 2007) was used to find benzene-metabolism-related genes. The search term “taxonomy:"Bacteria [2]” (benzene metabolism) AND reviewed: yes” was used, and the reviewed sequences were downloaded. Sequences for anaerobic benzene carboxylase (*ubiD*) (Atashgahi et al., 2018), periplasmic nitrate reductase (*napA*) (Schaedler et al., 2018), and aerobic toluene-4-monooxygenase (*tmoABCDEF*) (Atashgahi et al., 2018) were also retrieved from the database. As toluene-4-monooxygenase did not have a reviewed representative, the unreviewed sequences were used. Using MMseqs2 v. 12.113e3 (Steinegger and Söding, 2017), the sequences were collapsed using a 70% sequence identity cutoff to remove overrepresented protein sequences. The sequences were then aligned using Clustal Omega v. 1.2.4 (Sievers et al., 2011; Sievers and Higgins, 2017) and returned in Stockholm format using --*outfmt = st.* A hidden Markov model (HMM) was built from this file using the hmmbuild function of HMMER v. 3.2.1 (Eddy, 1998). The HMM was used to search the annotated contigs using the hmmsearch function of HMMER v. 3.2.1 (Eddy, 1998). Gene counts were normalized for total open reading frame number using OrfM v. 0.7.1 (Woodcroft et al., 2016). Assemblies were also analyzed for iron-cycling-related genes using FeGenie v. 1 (Garber et al., 2020). Assemblies were annotated using Prodigal v. 2.6.3 (Hyatt et al., 2010) contig_source *meta*. Gene counts were normalized using norm *y*.

Visuals for both HMM and FeGenie results were generated using ggplot2 v. 3.3.2 (Wickham, 2016), ggpubr v. 0.4.0 (Kassambara, 2020), and reshape v. 0.8.8 (Wickham, 2007). Gene count normalizations were converted to percentages for visualization using the package scales v. 1.1.1 (Wickham and Seidel, 2020). Color blind accessible palettes were applied to graphs using rcartocolor v. 2.0.0 (Nowosad, 2018).

### Sequence data availability

The 16S rDNA amplicon sequences are available at NCBI BioSample accession numbers SAMN39732271, SAMN39732272, SAMN39732273, SAMN39732274, SAMN39732275, SAMN39732276, SAMN39732277, SAMN39732278, SAMN39732279, and SAMN39732280. The metagenomic sequences are available at NCBI BioProject PRJNA1072096 with BioSample accession numbers SAMN39944932, SAMN39944933, SAMN39944934, SAMN33944935, SAMN33944936, SAMN33944937, SAMN33944938, SAMN33944939, SAMN39944940, and SAMN39944941. The “.fasta” files for each MAG assembly are available at https://kbase.us/n/105846/210/ via static narrative.

## Results

### Site conditions

Samples were collected over four sampling trips from one iron mat upstream and two iron mats downstream of a leaking underground storage tank seepage site in Town Creek, Greenville, NC (Figure 1). The upstream mat (U) served as a reference (unexposed) iron mat community within the system that was not impacted by the leaking underground storage tanks. Downstream, hydrocarbon-exposed mats (Da and Db) and water samples (W) were collected. Samples were collected twice in spring, March 2018 (S1 and S2), as well as two sampling efforts in summer, July (S3) and August (S4), 2019, to gain a broader understanding of microbial community dynamics over time.

### Geochemistry

**Figure 2:**
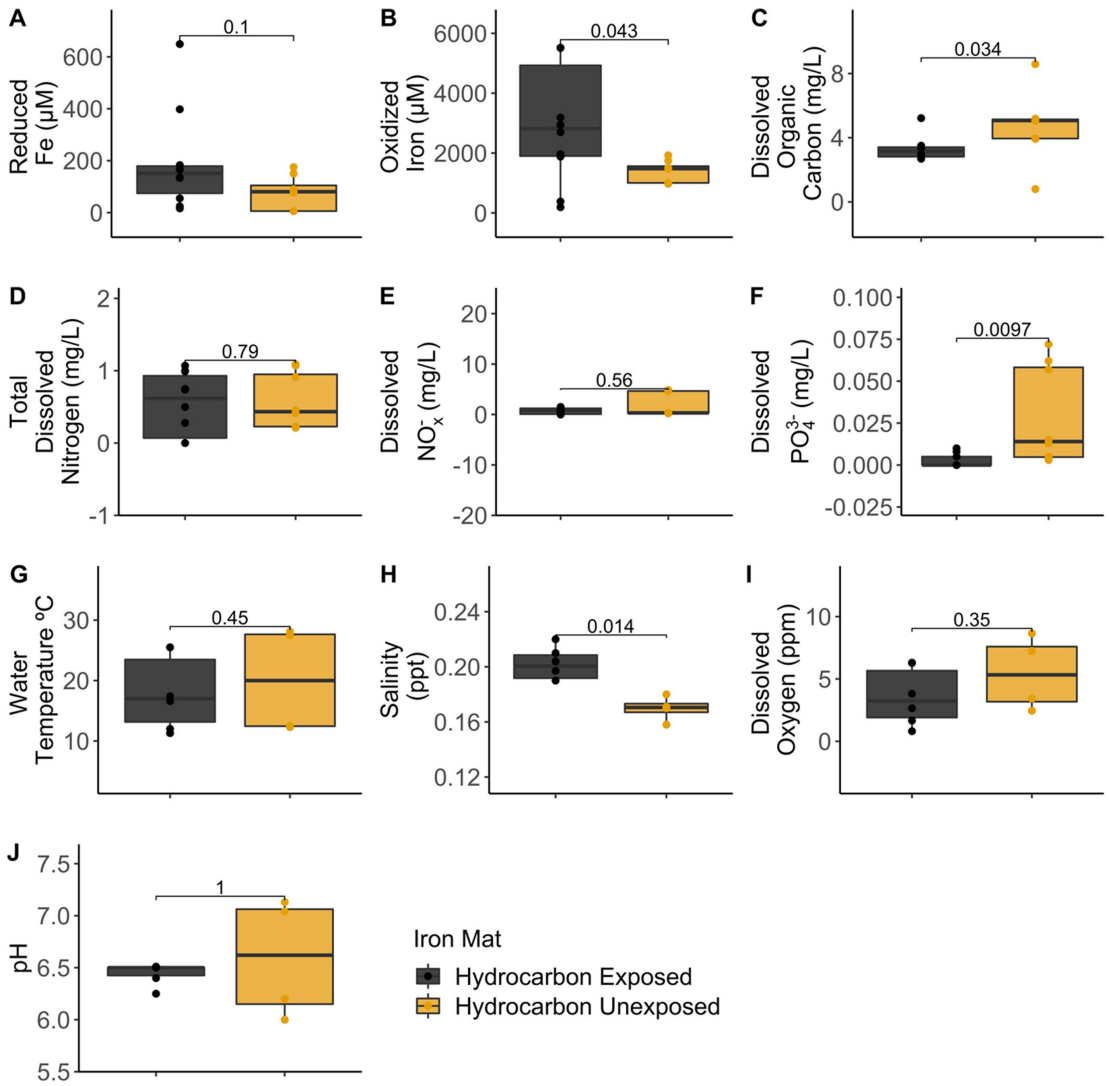
Comparisons between hydrocarbon-exposed (black) and unexposed (yellow) iron mats were made using Wilcoxon Rank Sum Tests for the measured analytes: **(A)** reduced iron (μM), **(B)** oxidized iron (μM), **(C)** dissolved organic carbon (mg/L), **(D)** total dissolved nitrogen (mg/L), **(E)** dissolved nitrates and nitrites (NO_x_^−^) (mg/L), **(F)** total dissolved phosphate (PO_4_^3−^) (mg/L), **(G)** water temperature (°C), **(H)** salinity (ppt), **(I)** dissolved oxygen (ppm), and **(J)** pH.

**Figure 3:**
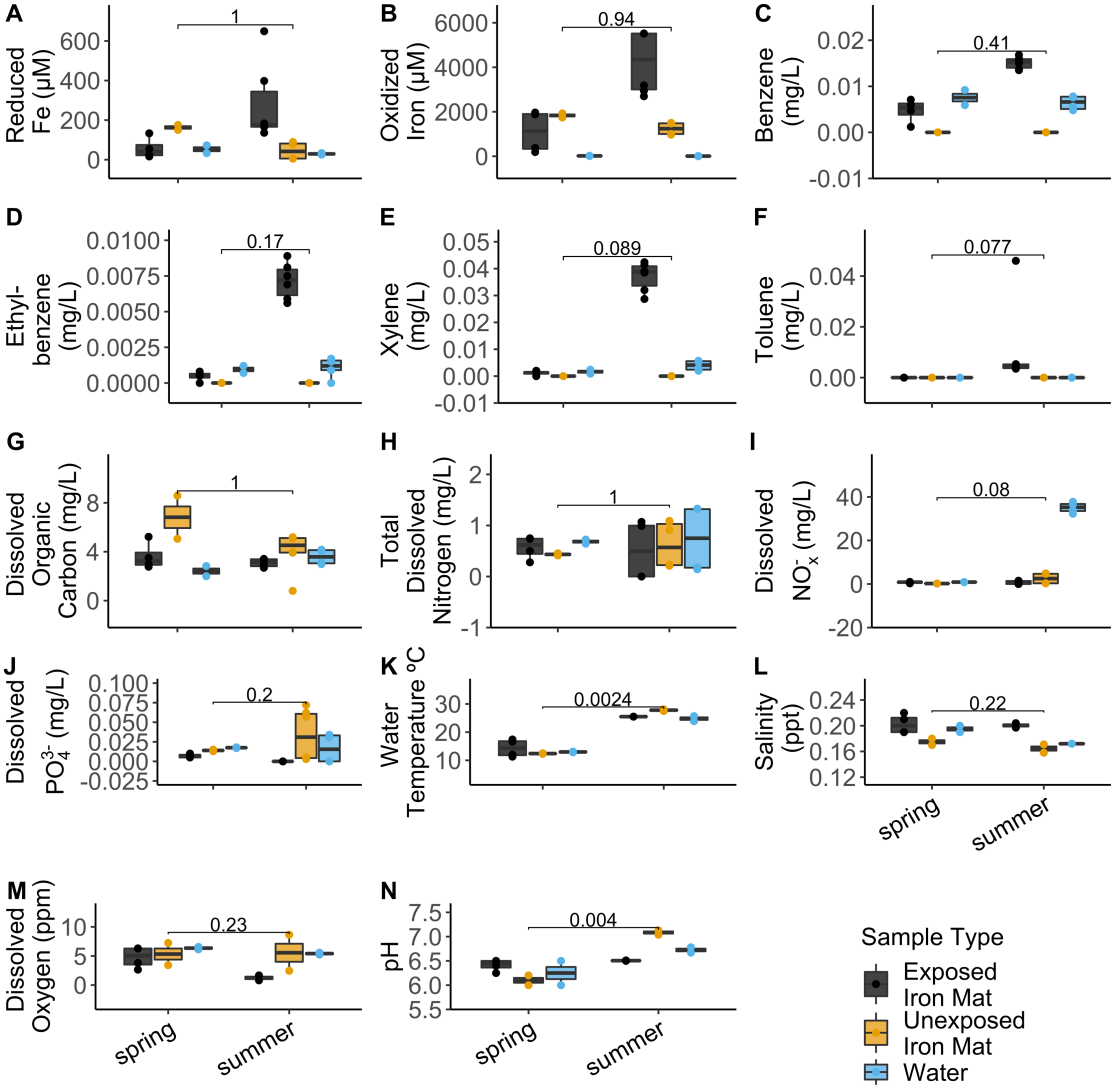
Comparisons for the measured analytes **(A)** reduced iron (μM), **(B)** oxidized iron (μM), **(C)** benzene (mg/L), **(D)** ethylbenzene (mg/L), **(E)** total xylenes (mg/L), **(F)** toluene (mg/L), **(G)** dissolved organic carbon (mg/L), **(H)** total dissolved nitrogen (mg/L), **(I)** dissolved nitrates and nitrites (NO_x_^−^) (mg/L), **(J)** total dissolved phosphate (PO_4_^3−^) (mg/L), **(K)** water temperature (°C), **(L)** salinity (ppt), **(M)** dissolved oxygen (ppm), and **(N)** pH between seasons (spring vs. summer) were made using Wilcoxon Rank Sum Tests. The results are displayed by sample type: hydrocarbon-exposed iron mat (black), unexposed iron mat (yellow), or hydrocarbon-exposed water sample (blue).

Analytical measurements from the unexposed and hydrocarbon exposed mats are shown in Supplementary Table S1. Comparisons between hydrocarbon-exposed and hydrocarbon-unexposed iron mats were made using Wilcoxon Rank Sum Tests. Hydrocarbon-exposed mats had significantly higher concentrations of oxidized iron (*p* = 0.043) and salinity (*p* = 0.014) than unexposed mats (Figure 2). Unexposed mats had significantly higher concentrations of dissolved organic carbon (*p* = 0.034) and dissolved PO_4_^3−^ (*p* = 0.009) (Figure 2). Comparisons between sampling seasons (spring vs. summer) were made using Wilcoxon Rank Sum Tests. Sampling season had a significant effect on water temperature (°C) (*p* = 0.002) and pH (*p* = 0.004) (Figure 3).

**Figure 4:**
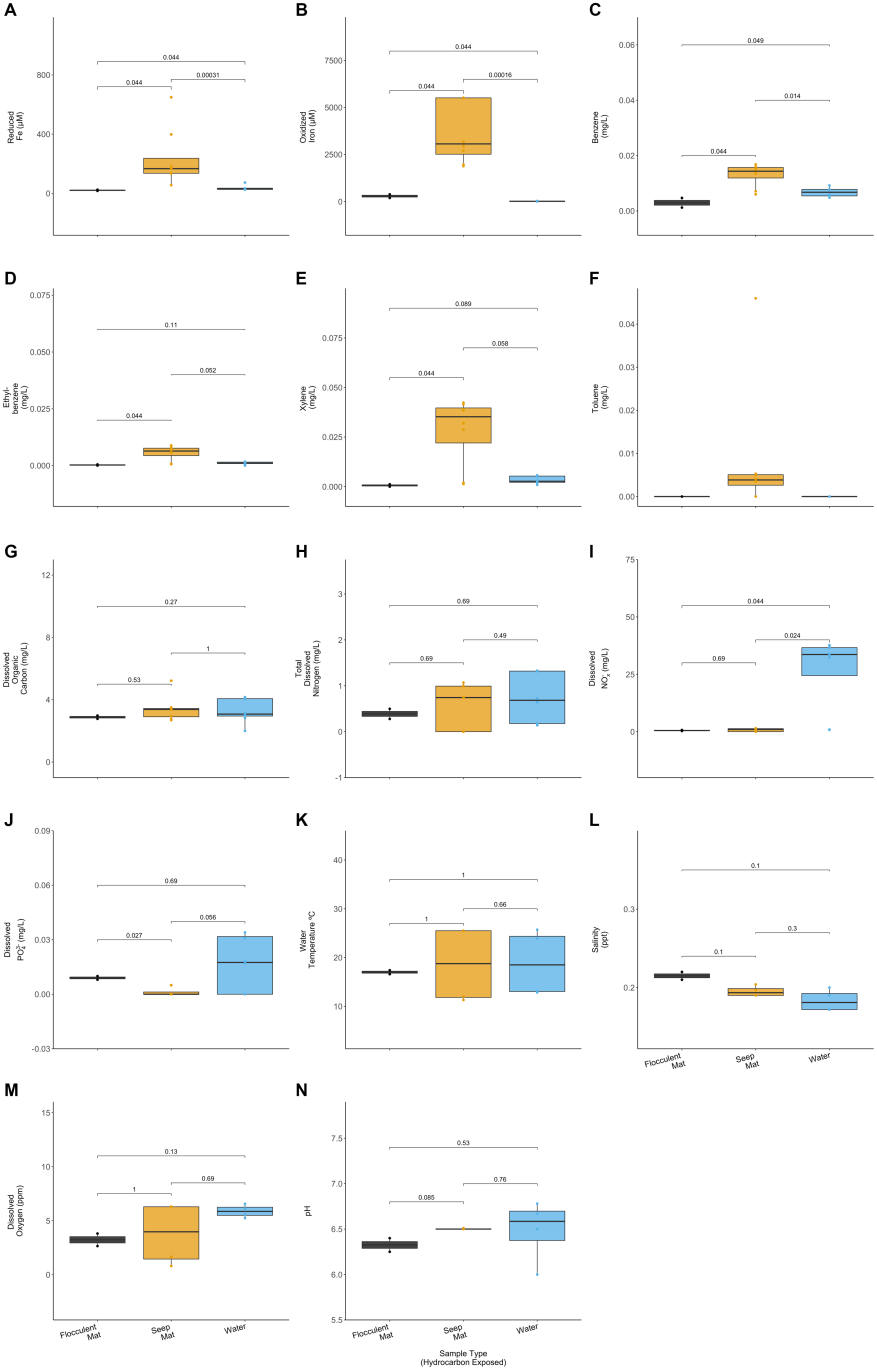
Hydrocarbon-exposed iron mats were designated to a mat type, either flocculent (black) or seep (yellow). Flocculent mats were in looser association and centimeters thick, whereas seep mats were denser and millimeters thick (Fleming et al., 2014). Mat types are plotted here with the hydrocarbon exposed water samples (blue) for **(A)** reduced iron (μM), **(B)** oxidized iron (μM), **(C)** benzene (mg/L), **(D)** ethylbenzene (mg/L), **(E)** total xylenes (mg/L), **(F)** toluene (mg/L), **(G)** dissolved organic carbon (mg/L), **(H)** total dissolved nitrogen (mg/L), **(I)** nitrates and nitrites (NO_x_^−^) (mg/L), **(J)** total dissolved phosphate (PO_4_^3−^) (mg/L), **(K)** water temperature (°C), **(L)** salinity (ppt), **(M)** dissolved oxygen (ppm), and **(N)** pH. Only two of the sampled iron mats were classifiable as flocculent, and both were sampled in spring. *p*-values for comparisons were calculated using Wilcoxon Ranked Sum Tests.

The hydrocarbon-exposed iron mats represented two previously observed mat types: flocculent and seep (Fleming et al., 2014). Flocculent mats are loosely associated and centimeters thick. Seep mats are densely associated and millimeters thick. All unexposed mats were seep mats and were not included in this statistical comparison due to their different geochemistry from exposed mats. Flocculent and seep mats had significantly different concentrations of reduced iron (*p* = 0.044), oxidized iron (*p* = 0.44), benzene (*p* = 0.044), ethylbenzene (*p* = 0.044), xylene (*p* = 0.044), and total dissolved PO_4_^3−^ (*p* = 0.027) (Wilcoxon Rank Sum Tests; Figure 4). Compared with the exposed water samples, flocculent and seep mats had significantly different concentrations of reduced iron (*p* = 0.044, 0.000), oxidized iron (*p* = 0.044, 0.000), benzene (*p* = 0.049, 0.014), and dissolved nitrates and nitrites (NO_x_^−^) (*p* = 0.044, 0.024) (Wilcoxon Rank Sum Tests; Figure 4). Benzene concentrations were observed to be lower in the flocculent mats than seep mats or water samples, and flocculent mats had higher dissolved PO_4_^3−^ concentrations than seep mats.

**Figure 5:**
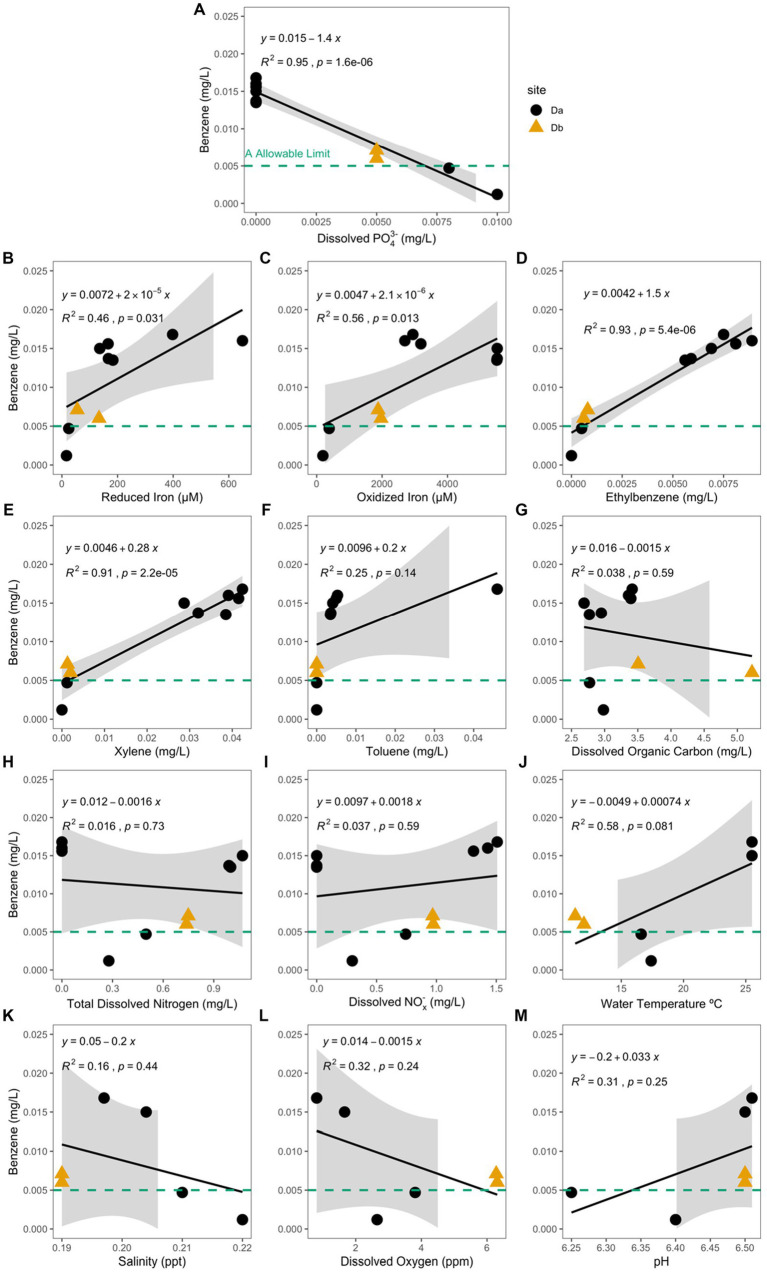
Benzene concentration (mg/L) at the hydrocarbon-exposed iron mat sampling sites, Da (black circle) and Db (yellow triangle) are plotted here against the measured analytes **(A)** total dissolved phosphate (PO_4_^3−^) (mg/L), **(B)** reduced iron (μM), **(C)** oxidized iron (μM), **(D)** ethylbenzene (mg/L), **(E)** total xylenes (mg/L), **(F)** toluene (mg/L), **(G)** dissolved organic carbon (mg/L), **(H)** total dissolved nitrogen (mg/L), **(I)** dissolved nitrates and nitrites (NO_x_^−^) (mg/L), **(J)** water temperature (°C), **(K)** salinity (ppt), **(L)** dissolved oxygen (ppm), and **(M)** pH. The EPA allowable limit for benzene (0.005 mg/L) is designated by a green-dashed line in each panel. Db was only measured during the first two time points (S1 and S2). Correlation and significance were calculated using a standard linear model, and the calculated equation is included on each panel. PO_4_^3−^ and benzene concentrations in the hydrocarbon-exposed iron mats have a significant negative correlation (*R*^2^ = 0.95, *p* = 1.6e^−6^), which is stronger than the correlation in the hydrocarbon-exposed water samples (not shown; *R*^2^ = 0.58, *p* = 0.028).

Of the analytes measured, concentration of dissolved phosphate (PO_4_^3−^) had the only significant negative correlation with the concentration of benzene (Pearson Correlation; *R*^2^ = 0.95, *p* = 1.6e^−6^; Figure 5A) and the hydrocarbon-exposed water samples (R^2^ = 0.58, *p* = 0.028). In the hydrocarbon-exposed iron mats, the concentration of reduced iron (R^2^ = 0.46, *p* = 0.031), oxidized iron (*R*^2^ = 0.56, *p* = 0.013), ethylbenzene (*R*^2^ = 0.93, *p* = 5.4e^−6^), and xylene (*R*^2^ = 0.91, *p* = 2.2e^−5^) had significant positive correlations with the concentration of benzene (Figure 5). In the hydrocarbon-exposed water samples, the concentration of reduced iron (*R*^2^ = 0.58, *p* = 0.027), ethylbenzene (*R*^2^ = 0.55, *p* = 0.035), and total dissolved nitrogen (*R*^2^ = 0.57, *p* = 0.03) had significant positive correlations with the concentration of benzene. In the hydrocarbon-exposed iron mats, PO_4_^3−^ also had a significant negative correlation with oxidized iron (*R*^2^ = 0.71, *p* = 0.0021), which was not observed in the hydrocarbon-exposed water samples (*R*^2^ = 0.24, *p* = 0.22) or unexposed iron mats (*R*^2^ = 0.13, *p* = 0.38).

### Community richness, diversity, and structure

Bacterial community composition was determined using operational taxonomic unit (OTU) relative abundances and was compared between exposed and unexposed iron mats. The alpha diversity indices (Supplementary Figure S1) and evenness index (Supplementary Figure S2) were significantly lower in exposed iron mats than unexposed iron mat microbial communities (e.g., Simpsons U = 0, *p* = 0.01421). Beta diversity did not significantly vary between exposed and unexposed iron mat microbial communities (ADONIS *R*^2^ = 0.12644, *p* = 0.234, strata = hydrocarbon exposure). Across seasons (spring vs. summer), alpha diversity (Supplementary Figure S3) and evenness (Supplementary Figure S4) were not significantly different. However, when evenness was modeled for both season and hydrocarbon exposure as factors, it was significantly different between seasons (*F* = 28.77, *p* = 0.001) and hydrocarbon exposure (*F* = 42.58, *p* = 0.0003). Beta diversity was also significantly different in iron mat communities when season was used as the stratum (ADONIS *R*^2^ = 0.25, *p* = 0.004, strata = season) (Supplementary Figure S5), and multiple OTUs were observed to be increased or decreased in abundance depending on season (Supplementary Figure S6).

**Figure 6:**
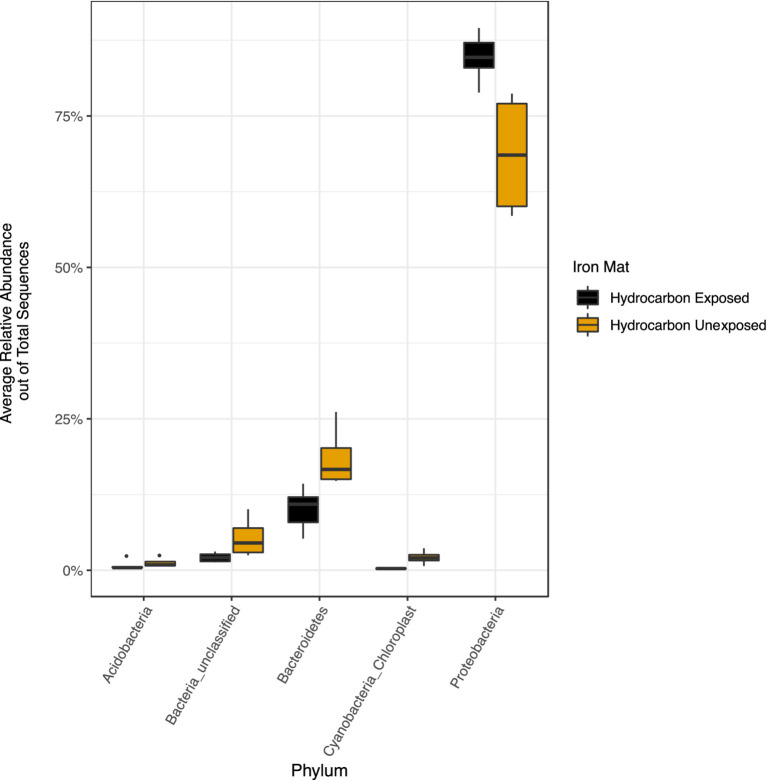
Relative abundances of phyla greater than 2 % were calculated out of total sequences for each sample and averaged by hydrocarbon exposure. Averages for present phyla are presented in yellow (unexposed) or black (hydrocarbon-exposed). The average relative abundance of all phyla except Proteobacteria are greater in unexposed samples. The increased relative abundance of Proteobacteria in the hydrocarbon-exposed iron mat microbial communities reflects the decreased overall diversity.

**Figure 7:**
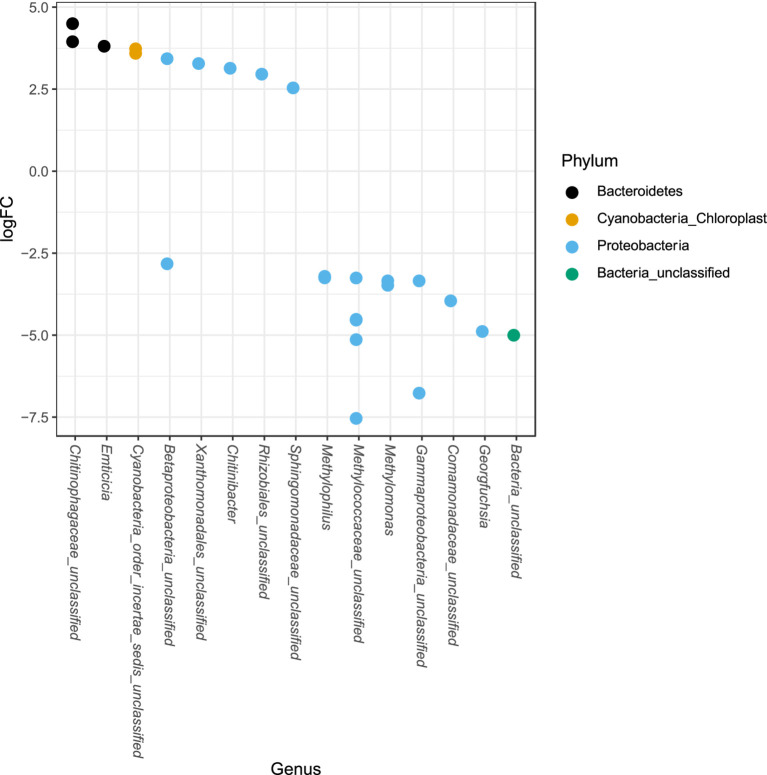
Differential abundances of taxa between upstream and downstream mats were calculated from an independently filtered data set. Genera with differential abundances with an alpha <0.05 were plotted. Each point on the plot represents a single OTU sequence. Points with a log-fold change greater than zero are of lower abundance in the hydrocarbon-exposed iron mat communities, whereas points with a log-fold change less than 0 are of increased abundance in hydrocarbon-exposed iron mat microbial communities relative to unexposed communities. The largest fold-changes were in OTUs in hydrocarbon-exposed communities and were observed in the highly represented phylum, Proteobacteria.

The phylum Proteobacteria had the greatest relative abundance among exposed (average 85%) and unexposed (average 69%) iron mat microbial communities, followed by Bacteroidetes (average 10 and 19%, respectively) (Figure 6). OTUs of significantly higher abundance were found using a log-fold change (logFC) analysis and were classified to be in the phyla Proteobacteria, Bacteroidetes, and Cyanobacteria (Figure 7). There were 11 OTUs with increased abundance in the unexposed iron mat microbial communities and 12 OTUs with increased abundance in the hydrocarbon-exposed iron mat microbial communities. The most highly abundant OTUs in the unexposed iron mats were classified to belong to the family Chitinophagaceae and the genus *Emticicia* (family Spirosomaceae). The most highly abundant OTUs in the hydrocarbon-exposed iron mats were classified to belong to the family Methylococcaceae and the class Gammaproteobacteria.

**Figure 8:**
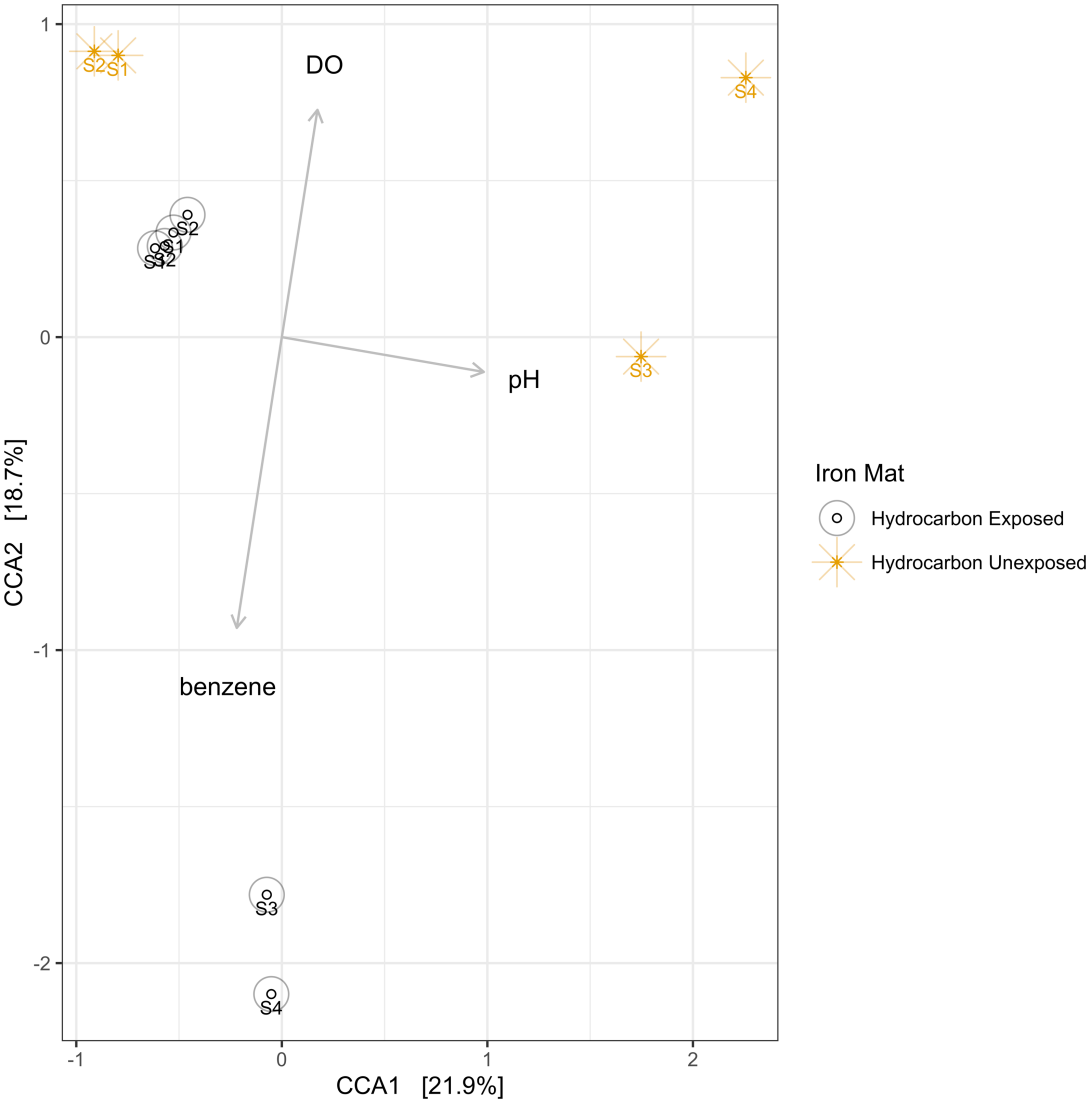
A canonical correspondence analysis (CCA) was used to calculate eigenvalues for the environmental conditions of iron mats. The overall model presented above is a good fit of the data (PERMANOVA *F* = 1.95, *p* = 0.002), and the variance inflation factor for each explanatory term is less than 3. The results indicate that the greatest effect on microbial community structure is from dissolved oxygen (DO; ppm), followed by pH and benzene concentration (mg/L). Sampling efforts (1–4) are labeled on each point.

Using a Canonical Correspondence Analysis (CCA), we observed that dissolved oxygen had the greatest statistical prediction power for community structure in the iron mats (PERMANOVA *F* = 2.61, *p* = 0.0033), followed by pH (PERMANOVA *F* = 2.22, *p* = 0.0192) and benzene concentration (PERMANOVA *F* = 1.06, *p* = 0.4326) (Figure 8). The vertical axis, represented by dissolved oxygen and benzene, together rendered the greatest separation between microbial communities from each unique sample. This separation was more apparent in summer than spring samples.

### Biogeochemical cycling potential—amplicon sequencing and metagenomes

The iron mat microbial community 16S amplicons were analyzed for taxa that potentially represent functional groups previously associated with iron mat biogeochemical cycling. These functional groups included iron-oxidizing bacteria, iron-reducing bacteria, sulfate-reducing bacteria, and nitrate-reducing bacteria. A special focus was also given to sequences that were classified as taxa that may be hydrocarbonoclastic bacteria (generally associated with the obligate use of hydrocarbons) or use benzene-degrading pathways.

Of the genera previously associated with microaerophilic iron-oxidation (Weiss et al., 2007; Fabisch et al., 2013; Kato et al., 2014; Fleming et al., 2018), only *Leptothrix* spp. were observed in the Town Creek iron mats by 16S rDNA amplicon sequence taxonomic classification (0.0095% relative abundance in hydrocarbon exposed mats, 0.0045% in unexposed mats). However, microscopy of iron mat samples revealed some FeOOH with a stalk morphology, which is associated with the stalk-forming *Gallionella* spp. (Fleming et al., 2014; Emerson et al., 2015; Gagen et al., 2018) These results, when paired, suggest that both stalk and sheath forming FeOB contribute to the production of iron mats in Town Creek.

Of the organisms that may play a role in anaerobic iron-oxidation, sequences for the putative nitrate-reducing iron-oxidizing bacteria in the genera *Acidovorax, Aquabacterium, Azospira, Paracoccus, Thermomonas,* and *Thiobacillus* (Hedrich et al., 2011) were recovered from both hydrocarbon-exposed and hydrocarbon-unexposed iron mats, with a higher relative abundance in hydrocarbon-exposed mats. Putative photoferrotrophic sequences were classified to the genera *Rhodobacter*, *Rhodomicrobium*, and *Rhodovulum* (Hedrich et al., 2011). These sequences had a higher relative abundance in unexposed iron mats.

On average, sequences classified as genera associated with FeRB (Supplementary Table S2) totaled 0.755 and 0.971% relative abundance of sequences from hydrocarbon-exposed and unexposed iron mats, respectively. Of these, the genus *Geobacter* has the potential to couple iron reduction with benzene degradation (Tremblay and Zhang, 2020) and represented an average of 0.56 and 0.74% relative abundance of hydrocarbon-exposed and unexposed iron mats, respectively. Sequences classified as genera associated with SRB (Supplementary Table S3), on average, 0.143 and 0.209% relative abundance of hydrocarbon-exposed and hydrocarbon-unexposed iron mats, respectively. Amplicon sequences were classified as the genus *Desulfobacula*, which may couple sulfate reduction with benzene degradation (Chakraborty and Coates, 2004), averaged 0.0026 and 0.0010% relative abundance of hydrocarbon-exposed and hydrocarbon-unexposed iron mats. Amplicon sequences classified as the genera *Hydrogenophaga* and *Dechloromonas*, which have the potential to couple nitrate reduction to benzene degradation (Coates et al., 2001; Fahy et al., 2006, 2008), were observed in hydrocarbon-exposed (average 5.27 and 0.16%, respectively) and hydrocarbon-unexposed (average 3.96 and 0.19%, respectively) iron mats. Only one genus of hydrocarbonoclast, *Planomicrobium,* was represented in classified amplicon sequences. *Planomicrobium* had a greater relative abundance in unexposed iron mat communities; however, they accounted for a very small relative abundance of these sequences (<0.0025%) from any sample.

Assembled contigs from the metagenomic sequences were assessed for quality (Supplementary Table S4) and then assigned functional potential using HMM analysis. There was no significant difference between hydrocarbon-exposed and unexposed sequences for iron-cycling (e.g., *cyc1, cyc2, mtrB, and mtoA*) or benzene-remediation (e.g., *napA, ubiA, fdnGHI, and pcrA*) gene sequence-normalized abundance (Supplementary Figures S7, S8).

### Recovered MAGs

Twenty-nine MAGs were recovered from the iron mat samples (> 59% complete, < 10% contamination) (Supplementary File S3). Hydrocarbon exposure did not have an observable effect on genome size (unexposed average 2.57 ± [standard error = SE] 0.24 Mbp; hydrocarbon exposed average 2.61 ± SE 0.13 Mbp) or GC content (unexposed average 52.67 ± SE 4.68%; hydrocarbon exposed average 55.4 ± SE 2.17%). Greater taxonomic diversity in the MAGs was recovered from the unexposed iron mats, including two MAGS that were classified as the protist endosymbiont, *Phycorickettsia* spp. (Yurchenko et al., 2018). Seven MAGs (one unexposed/six hydrocarbon-exposed) were classified as belonging to the genus *Gallionella* (avgerage genome completeness 96%, contamination 2%). Eight MAGs (one unexposed/seven hydrocarbon-exposed) were classified as belonging to the family Burkholderiaceae (avgerage genome completeness 93%, contamination 3%). Seven of the eight Burkholderiaceae MAGs, excluding only MAG #13, had genomic evidence of iron oxidation genes (*cyc1*, *cyc2*, *mtrB*, and *mtoA*) based on the FeGenie analysis (Supplementary Table S5).

Whole genome representatives of FeOB from NCBI including *Mariprofundus ferrooxydans* PV-1, *Mariprofundus erugo*, *Gallionella capsiferriformans* ES-2, *Ferriphaselus amnicola*, and *Leptothrix ochracea* (Supplementary File S4) were annotated using DRAM, as well as the Burkholderiaceae family (Supplementary File S5) and *Gallionella* spp. (Supplementary File S6) MAGs. Of the isolated representatives of microaerophilic FeOB, *Leptothrix ochracea* was the most dissimilar, missing modules for the Entner-Doudoroff and reductive acetyl-CoA pathways, and ETC complexes NADH: quinone oxidoreductase, succinate dehydrogenase, and cytochrome bd ubiquinol oxidase. *L. ochracea* was also missing indicators of carbohydrate-active enzymes (CAZY), methanogenesis, and short-chain fatty acid metabolisms that were present in all other FeOB isolates. Of those MAGs assigned to the family Burkholderiaceae, MAG 26 was missing module for the Entner–Duodoroff and reductive acetyl-CoA pathways, as well as the CAZY, methanogenesis, and short-chain fatty acid metabolism indicators missing from the *L. ochracea* genome. All eight MAGs classified as Burkholderiaceae were missing from the ETC complex succinate dehydrogenase. Of the MAGs assigned to the genus Gallionella, all seven MAGs were missing modules for the Entner–Duodoroff pathway, as well as the ETC complexes for succinate dehydrogenase and cytochrome bd ubiquinol oxidase.

## Discussion

This study of the freshwater iron mat system begins to address how contaminant hydrocarbon exposure influences geochemistry, microbial community structure, and potential microbial function. We found that hydrocarbon exposure had a significant effect on iron mat-oxidized iron, dissolved organic carbon, and dissolved PO_4_^3−^, independent of seasonal effects. Organic carbon concentration has been previously associated with *Leptothrix ochracea*-dominated iron mats (Fleming et al., 2014), such as those in Town Creek. The higher observed dissolved organic carbon in the unexposed iron mats helps to explain the higher number of recovered *Gallionella* spp. MAGs from the hydrocarbon exposed iron mats, where conditions likely were less favorable to *Leptothrix ochracea*.

Our amplicon sequencing results showed the presence of photoferrotrophs in the iron mats. The photoferrotrophs had a higher relative abundance in the unexposed iron mats, which may reflect the decreased irradiance in water exposed to hydrocarbons from the leaking underground storage tanks. In hydrocarbon-contaminated water, there was a thin sheen of both oxidized iron (“schwimmeisen”) and oil floating on the top of the water, potentially decreasing niche suitability for phototrophic organisms. This was further supported as the logFC analysis suggested that Cyanobacteria were over-expressed in the unexposed iron mat microbial communities compared with the hydrocarbon-exposed communities.

The presence of nitrate-reducing iron-oxidizing taxa was also suggested by the amplicon sequencing results. Previously, three of these genera (*Azospira, Paracoccus,* and *Thermomonas*) have not been found to be present in freshwater iron mat microbial communities (Brooks and Field, 2020); however, *Azospira* has been identified previously in paddy soil (Li et al., 2016). These OTUs had higher relative abundances in the hydrocarbon-exposed iron mats. The exposed iron mats also had significantly lower NO_x_^−^ concentrations than the exposed water samples. The lower concentration of NO_x_^−^ did not have a significant correlation with any of the hydrocarbons measured. We hypothesize that active denitrification processes could be concentrated within the iron mats exposed to hydrocarbons. This may prove to be an important connection between the nitrogen cycle and freshwater iron mats, a connection that is only just being explored in marine iron mats (McAllister et al., 2021; Hribovšek et al., 2023). Alternatively, it has been observed in terrestrial samples that reduced iron reacts with nitrate to form Fe(OH)_3_ and N_2_O (Matus et al., 2019).

We observed a strong negative correlation between dissolved PO_4_^3−^ and benzene in hydrocarbon exposed iron mats. This result, paired with significantly higher benzene concentrations in seep mats than the exposed water samples, suggests the differing geochemistry between iron mats and the surrounding water column likely influences hydrocarbon behavior. The concentration of dissolved PO_4_^3−^ was also significantly greater in unexposed iron mats. Previously, FeOOH in iron mats has been demonstrated to adsorb phosphorous (Takeda et al., 2010; Buliauskaitė et al., 2020). We hypothesize that benzene and PO_4_^3−^ interact antagonistically within the system, leading to competitive exclusion from the surface of FeOOH. Alternatively, the addition of phosphorous has been previously observed to increase microbial benzene removal (Xiong et al., 2012). Higher phosphorous concentrations may increase microbial scavenging of benzene, leading to a strong negative correlation. Further studies are needed to understand the negative correlation we observed between phosphate and benzene, and whether it is chemically, physically, or biologically driven. We recommend future experimental studies on the use of freshwater iron mats that killed controls with added phosphate, nitrates, and nitrites and organic carbon to better disentangle the chemical reactions in iron mat environments from biological reactions.

Alpha diversity and evenness were observed to be significantly lower in the hydrocarbon-exposed than unexposed iron mat microbial communities, which suggests that the within community diversity of iron mats is impacted by contaminant exposure. This is consistent with other oil-exposed microbial communities (Maila et al., 2005; Máthé et al., 2012). We also observed a potential interaction between season and hydrocarbon exposure. Evenness was higher in iron mat communities in summer compared with spring. A similar trend has been shown in riverine microbial communities to be associated with changes in flow resulting from seasonal precipitation differences (Luo et al., 2020) or hydrological factors, such as groundwater discharge (Valett et al., 1997). Seasonal differences have also been previously observed in the microbial communities of freshwater ion mats (Fleming et al., 2014). The Town Creek iron mat samples, however, did not have the confounding variable of dominant microaerophilic FeOB, which was observed in the previous study, to change with the seasons (Fleming et al., 2014). A previous study has found that the concentration and type of hydrocarbon present in watersheds in Mexico varied with season (spring and summer) are attributed to increased precipitation and motor traffic (Narciso-Ortiz et al., 2020). While we did not identify differences in the types of hydrocarbons present in the downstream mats by season, the concentrations of benzene, ethylbenzene, and xylenes were all higher in summer (0.015 ± 0.001 mg/L, 0.007 ± 0.000 mg/L, and 0.037 ± 0.005 mg/L) than spring (0.005 ± 0.003 mg/L, 0.000 ± 0.000 mg/L, and 0.001 ± 0.001 mg/L).

Both hydrocarbon-exposed and unexposed iron mat microbial communities were dominated by the phylum Proteobacteria. This is consistent with other studies of hydrocarbon-exposed microbial communities; however, the samples from previous studies tend to be dominated by Alphaproteobacteria (Saul et al., 2005; Zhao et al., 2020; Eze, 2021), whereas the iron mat sample 16S rDNA amplicon sequences (regardless of hydrocarbon exposure) were dominated by Betaproteobacteria (Supplementary Figure S9). The relative abundance of Betaproteobacteria, which may be associated with the degradation of lower molecular weight polycyclic aromatic hydrocarbons (Sun et al., 2019), was observed to be higher in hydrocarbon-exposed than in unexposed iron mats. These observations suggest that iron mat microbial community diversity and composition are impacted by exposure to hydrocarbons.

Only one genus of hydrocarbonoclast was observed at Town Creek: *Planomicrobium*. This is in agreement with previous findings in cyanobacterial mats that hydrocarbonoclastic genera are not good indicators of chronic hydrocarbon exposure; rather, they are cosmopolitan (Aubé et al., 2016). There was, however, a greater relative abundance of 16S rDNA amplicon OTUs associated with nitrate reduction-coupled benzene degradation (Coates et al., 2001; Fahy et al., 2006, 2008) in the hydrocarbon-exposed iron mats. Other OTUs classified as genera associated with iron reduction and sulfate reduction coupled to benzene degradation were observed in both hydrocarbon-exposed and unexposed iron mat microbial communities but at much lower relative abundances. These result in total represent possible connections between hydrocarbons and the nitrogen, iron, and sulfur cycles and suggest that the community within the iron mat could potentially contribute to bioremediation of benzene or other hydrocarbons. Freshwater iron mats have been previously hypothesized to connect the iron and sulfur cycles (Koeksoy et al., 2018; Brooks and Field, 2021); however, connections to the nitrogen cycle have not been previously proposed. Of interest, future studies would be whether microbial activity rates in the iron mat are the same when exposed to hydrocarbons, given the previously discussed loss of diversity.

Using a canonical correspondence analysis, it was observed that dissolved oxygen and pH were significantly correlated with microbial community structure. Iron mats are assemblages of microorganisms sensitive to oxygen conditions: microaerophiles, anaerobes, and aerobes. The microbial communities are therefore likely to be sensitive to changes in dissolved oxygen. Dissolved oxygen had a greater influence on community structure in the summer, reflecting a very low average dissolved oxygen concentration in hydrocarbon-exposed iron mats (average 2.0 ppm). Previous studies have shown that pH is a strong driver of community structure, even in communities exposed to polycyclic aromatic hydrocarbons (Wu et al., 2017). However, this result is somewhat surprising given that the pH of this data set only ranged from 6 to 7.13, which likely falls within the growth range of many microorganisms. Perhaps this result reflects the influence of pH on the biogeochemical cycles that were catalyzed or vice versa. It was also observed that functional potential, which was found using hidden Markov models for iron-cycling and benzene-remediation genes, did not significantly vary between hydrocarbon exposure states. This is inconsistent with a previous study of marine sediment core communities that find an increase in the potential of iron-cycling under hydrocarbon regimes (Zhao et al., 2020). In contrast, our results reflect iron-cycling as a key process in iron mats, which is unlikely to change under contaminant exposure. These results highlight that microbial communities exposed to contaminants are also under common selective pressures and reflect other environmental factors.

We were able to recover 29 MAGs from the iron mat microbial communities. MAGs are highly useful for linking geochemical cycles within a single taxon by identifying how they contribute holistically. MAGs have been found to be useful in identifying rare taxa from data sets that are not previously observed using 16S rDNA amplicon sequencing (Wilkins et al., 2019). None of the recovered MAGs were assigned to taxa known for hydrocarbon degradation. Seven MAGs (one unexposed/six hydrocarbon exposed) were classified to the genus of microaerophilic iron-oxidizing bacteria, *Gallionella*. The DRAM annotation of these MAGs was dissimilar to the annotation of the sequenced representative, *Gallionella capsiferriformans* ES-2. All seven MAGs were missing modules for the Entner–Duodoroff pathway (catabolizing glucose to pyruvate), as well as ETC complexes for succinate dehydrogenase (citric acid cycle/electron transport chain) and cytochrome bd ubiquinol oxidase (reduction of molecular oxygen). While it is possible that these modules are missing due to incomplete binning, previous study has demonstrated that MAGs with >90% completeness should be effective representation of organismal functions (Nelson et al., 2020). Two MAGs from the unexposed iron mat metagenomes were classified as the endosymbiont *Phycorickettsia* spp. The presence of these endosymbionts from MAGs indicates the putative presence of protists or other microeukaryotes in the upstream iron mats that would host these organisms, though studies of microeukaryotic members of the iron mat community are currently in need of further research (Brooks and Field, 2020).

This study aimed to pair geochemical and molecular data to develop an understanding of how contaminant exposure, specifically hydrocarbons, would impact freshwater iron mat geochemistry, microbial community structure, and microbial community function. We found significant effects of hydrocarbon exposure on iron mat geochemistry and microbial community diversity. We observed an especially interesting correlation between the concentrations of benzene and dissolved phosphate. We also observed key differences between iron mat types (seep vs. flocculent) in concentrations of reduced and oxidized iron, phosphate, and hydrocarbons. Our results suggested that some functional groups, detected using 16S rDNA amplicon sequencing, vary in the relative abundance correlated with hydrocarbon exposure. However, gene detection using hidden Markov models on metagenomic contigs did not demonstrate significant changes in iron-cycling or benzene-degrading functional potential. Previous research in other ecosystems has demonstrated links between community diversity shifts and changes in functional potential in microbial communities (Galand et al., 2018). We hypothesize that it may be possible to observe changes in activity in our system if alternative methods (i.e., direct microbial metabolism measurements or RT-qPCR) are applied in future studies. Additionally, increasing sequencing depth for future metagenomic analyses of these freshwater iron mats may also provide more functional data and help identify more differences between exposed and unexposed iron mat communities. However, these results are encouraging to research on iron mats, providing a good foundation, especially toward application in systems that are currently unbuffered from hydrocarbon pollution. With over 500,000 leaking storage tanks in the United States, it is inevitable that these pollutants will reach above ground reservoirs, where, as indicated here, the iron mat microbial community may be successfully applied at that oxic–anoxic interface.

## Data availability statement

The 16S rDNA amplicon sequences are available at NCBI BioSample Accession numbers SAMN39732271, SAMN39732272, SAMN39732273, SAMN39732274, SAMN39732275, SAMN39732276, SAMN39732277, SAMN39732278, SAMN39732279, and SAMN39732280. The metagenomic sequences are available at NCBI BioProject PRJNA1072096 with BioSample Accession numbers SAMN39944932, SAMN39944933, SAMN39944934, SAMN33944935, SAMN33944936, SAMN33944937, SAMN33944938, SAMN33944939, SAMN39944940, and SAMN39944941. The “.fasta” files for each MAG assembly is available at https://kbase.us/n/105846/210/ via static narrative.

## Author contributions

CB: Conceptualization, Data curation, Formal analysis, Funding acquisition, Investigation, Methodology, Writing – original draft, Writing – review & editing. EF: Conceptualization, Formal analysis, Investigation, Methodology, Project administration, Writing – review & editing.

## Footnotes

^1^ https://mothur.org/wiki/miseq_sop/

^2^ https://joey711.github.io/phyloseq-extensions/edgeR.html
