# Supplementary material for: Microbial community response to hydrocarbon exposure in iron oxide mats: an environmental study
Source: Front Microbiol. 2024 May 10;15:1388973. doi: 10.3389/fmicb.2024.1388973 (PMC11116660; doi:10.3389/fmicb.2024.1388973)
Supplement: Supplementary file 3 [file Data_Sheet_1.PDF]

Microbial Community Response to Hydrocarbon Exposure in Iron Oxide Mats: An Environmental Study

Chequita N. Brooks[1,2] and Erin K. Field[1*]

[1]Department of Biology, East Carolina University, Greenville, NC, USA
[2]Louisiana Universities Marine Consortium, Chauvin, LA, USA

Supplemental Information

Supplemental Table 1: Geochemical conditions from each site at Town Creek, Greenville, NC. Iron mats were categorized by location as upstream (U; unexposed), downstream A (Da; hydrocarbon exposed), or downstream B (Db; hydrocarbon exposed). Samples for geochemical analysis were also collected from a hydrocarbon exposed water sample (W). Measurements are displayed as averages ± standard deviation across all timepoints. Raw data can be found in Supplemental File 2.

**Site**	**Upstream (U)**	**Downstream A (Da)**	**Downstream B (Db)**	**Water (W)**
Reduced Iron (µM)	74.199 ± 23.324	217.518 ± 74.354	94.210 ± 39.136	35.291 ± 5.494
Oxidized Iron (µM)	1388.361 ± 128.057	3242.853 ± 772.530	1922.800 ± 43.313	10.537 ± 2.370
Total Iron (µM)	1462.560 ± 150.656	3460.372 ± 787.535	2017.010 ± 82.450	45.828 ± 6.494
Benzene (mg/L)	0.000 ± 0.000	0.012 ± 0.002	0.007 ± 0.001	0.0067 ± 0.0006
Ethylbenzene (mg/L)	0.000 ± 0.000	0.0054 ± 0.0012	0.0007 ± 0.0001	0.001 ± 0.000
Xylene (mg/L)	0.000 ± 0.000	0.028 ± 0.006	0.002 ± 0.000	0.003 ± 0.001
Toluene (mg/L)	0.000 ± 0.000	0.008 ± 0.005	0.000 ± 0.000	0.000 ± 0.000
Dissolved Organic Carbon (NPOC) (mg/L)	4.717 ± 0.758	3.042 ± 0.108	4.363 ± 0.858	3.292 ± 0.272
Total Nitrogen (TN) (mg/L)	0.575 ± 0.136	0.480 ± 0.170	0.743 ± 0.005	0.728 ± 0.190
Nitrates & Nitrites (mg/L)	1.947 ± 0.818	0.661 ± 0.238	0.972 ± 0.003	26.554 ± 5.643
Total Dissolved Phosphate (PO_4) (mg/L)	0.0023 ± 0.0015	0.0023 ± 0.0019	0.0050 ± 0.0000	0.0168 ± 0.0054
Water Temperature (ºC)	20.10 ± 4.45	21.25 ± 2.46	11.65 ± 0.35	18.89 ± 3.43
Air Temperature (ºC)	17.4 ± 6.8	17.4 ± 6.8	5.8 ± 2.8	17 ± 7.2
Salinity (ppt)	0.170 ± 0.005	0.208 ± 0.005	0.190 ± 0.000	0.183 ± 0.007
Dissolved Oxygen (DO) (mg/L)	5.443 ± 1.490	2.233 ± 0.646	6.295 ± 0.015	5.880 ± 0.293
pH	6.6 ± 0.3	6.4 ± 0.1	6.5 ± 0.0	6.5 ± 0.2

Supplemental Table 2: 16S rDNA amplicon sequencing OTUs from iron mat samples were assigned to genera of iron-reducing bacteria. Averages of the percent relative abundance of each genera from hydrocarbon exposed iron mats are presented below. References used to hypothesize function of genera as iron-reducers are in the "Reference" column.

Genus	Hydrocarbon Exposed Average (% relative abundance)	Unexposed Average (% relative abundance)	Reference
Geobacter	0.558	0.738	(1)
Shewanella	0.002	0.011	(1)
Rhodoferax	0.004	0.006	(1)
Geobacteraceae (family – unclassified genus)	0.0005	0	(1)
Geothrix	0.179	0.211	(2)
Desulfuromonas	0.011	0.005	(3)

Supplemental Table 3: 16S rDNA amplicon sequencing OTUs from iron mat samples were assigned to genera of sulfate-reducing bacteria. Averages of the percent relative abundance of each genera from hydrocarbon exposed iron mats are presented below. References used to hypothesize function of genera as sulfate-reducers are in the "Reference" column.

Genus	Hydrocarbon Exposed Average (% Relative Abundance)	Unexposed Average (% Relative Abundance)	Reference
Desulfobacter	0.0005	0	(4)
Desulfobacterium	0.002	0.004	(4)
Desulfobacula	0.003	0.001	(5)
Desulfobulbus	0.023	0.049	(5)
Desulfomonile	0.001	0.022	(4)
Desulfosporosinus	0.007	0.0007	(5)
Desulfovibrio	0.032	0.012	(5)
Thermodesulfovibrio	0.003	0.003	(5)
Desulfomicrobium	0.006	0.024	(5)
Desulfobacteraceae (family – unclassified genus)	0.013	0.037	(5)
Desulfobacterales (family – unclassified genus)	0.025	0.017	(5)
Desulfuromonadales (family – unclassified genus)	0.008	0.019	(6)
Desulfatirhabdium	0.01	0.009	(7)
Desulforegula	0.005	0.0007	(8)
Desulfatiferula	0.002	0.001	(9)
Desulfopila	0.001	0.0003	(10)
Desulfovibrionales (family – unclassified genus)	0	0.001	(5)
Desulfovibrionaceae (family – unclassified genus)	0.0004	0	(11)
Syntrophobacteer	0.0006	0.008	(12)

Supplemental Table 4: Metagenome assembly quality statistics calculated using the MetaQUAST function in QUAST v. 5.0.2 (19). The interpretation of each value can be read about in the QUAST manual (http://quast.sourceforge.net/docs/manual.html; Accessed 2021JAN26). Briefly, the "Genome Fraction (%)" is the percentage of aligned bases to the reference genome. As no reference genome was provided, MetaQUAST called BLASTN to align contigs to the SILVA 16S rRNA database and the 50 reference genomes with the top alignment scores were chosen.

Sample	Genome Fraction (%)	# Misassemblies	# Contigs	# Contigs ≥ 1,000 bp	# Contigs ≥ 50,000 bp	Largest Contig (bp)	Total Length (bp)	N50	L50
U1	78.804	10	73,668	15,862	1	56,329	70,497,637	929	18,194
U2	0.248	11	94,743	15,437	5	68,160	81,932,399	796	26,850
U3	0.419	20	146,638	26,524	32	357,575	134,208,763	855	38,458
U4	0.173	14	123,682	29,277	21	357,707	123,454,918	1017	28,398
Da1	56.622	3	116,521	20,427	12	131,329	103,617,361	827	32,208
Da2	22.942	22	29,106	4,644	5	96,818	26,093,544	813	7,344
Da3	34.084	13	142,409	32,539	49	452,140	145,282,894	1,009	31,954
Da4	37.425	11	163,329	33,808	116	587,965	166,293,253	975	35,766
Db1	21.919	13	76,123	16,525	14	141,907	76,414,582	980	16,905
Db2	23.388	23	90,221	22,283	24	174,066	92,447,762	1050	20,501
**Average± Standard Error**	27.60 ±8.11	14 ±1.94	105,644 ±12,872.08	21,687.3 ±2872.78	27.9 ±10.80	242,399.6 ± 58,046.75	102,024,311 ± 13,085,240	925.1 ± 29.88	25,657.8 ± 3,076.44

Supplemental Table 5: Here we present the MAGs that were classified by MetaSanity as being in the family Burkholderiaceae, with each ✓ representing an open reading frame in that MAG as being the gene in the first row. MAGS were searched for iron oxidation genes using the HMMs in FeGenie (18). Possible iron oxidation genes with HMMs are *cyc1*, *cyc2*_repCluster1, *cyc2*_repCluster2, *cyc2*_repCluster3, *foxA*, *foxB*, *foxC*, *foxE*, *foxY*, *foxZ*, *mtoA*, *mtrB*_TIGR03509, and suflocyanin.

**MAG #**	***cyc1***	***cyc2*_repCluster1**	***mtrB*_TIGR03509**	***mtoA***
**3**		✓		
**13**				
**15**	✓	✓	✓	✓
**16**	✓	✓		
**18**	✓	✓		
**22**	✓	✓		
**26**	✓	✓		
**28**	✓	✓		

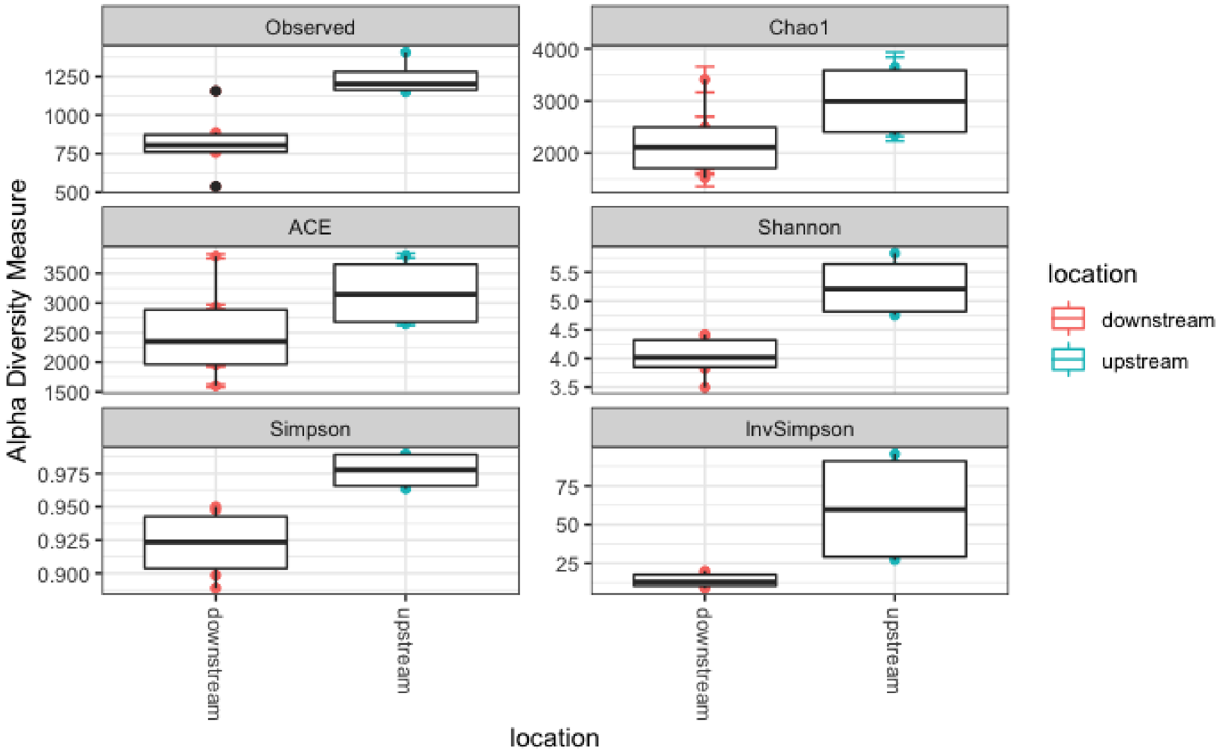

Supplemental Figure 1: Alpha diversity indices for hydrocarbon exposed (downstream) and unexposed (upstream) iron mat microbial communities were compared using a Mann-Whitney-Wilcoxon test in R v. 3.5.2. Observed Species Richness (U = 1, p = 0.02518), Shannon Diversity Index (U = 0, p = 0.01421), Simpsons Diversity Index (U = 0, p = 0.01421), and Inverse

Simpsons Diversity Index (U = 0, p = 0.1421) were all significantly different between upstream and downstream iron mats. Also plotted are Chao1 and ACE alpha diversity indices.

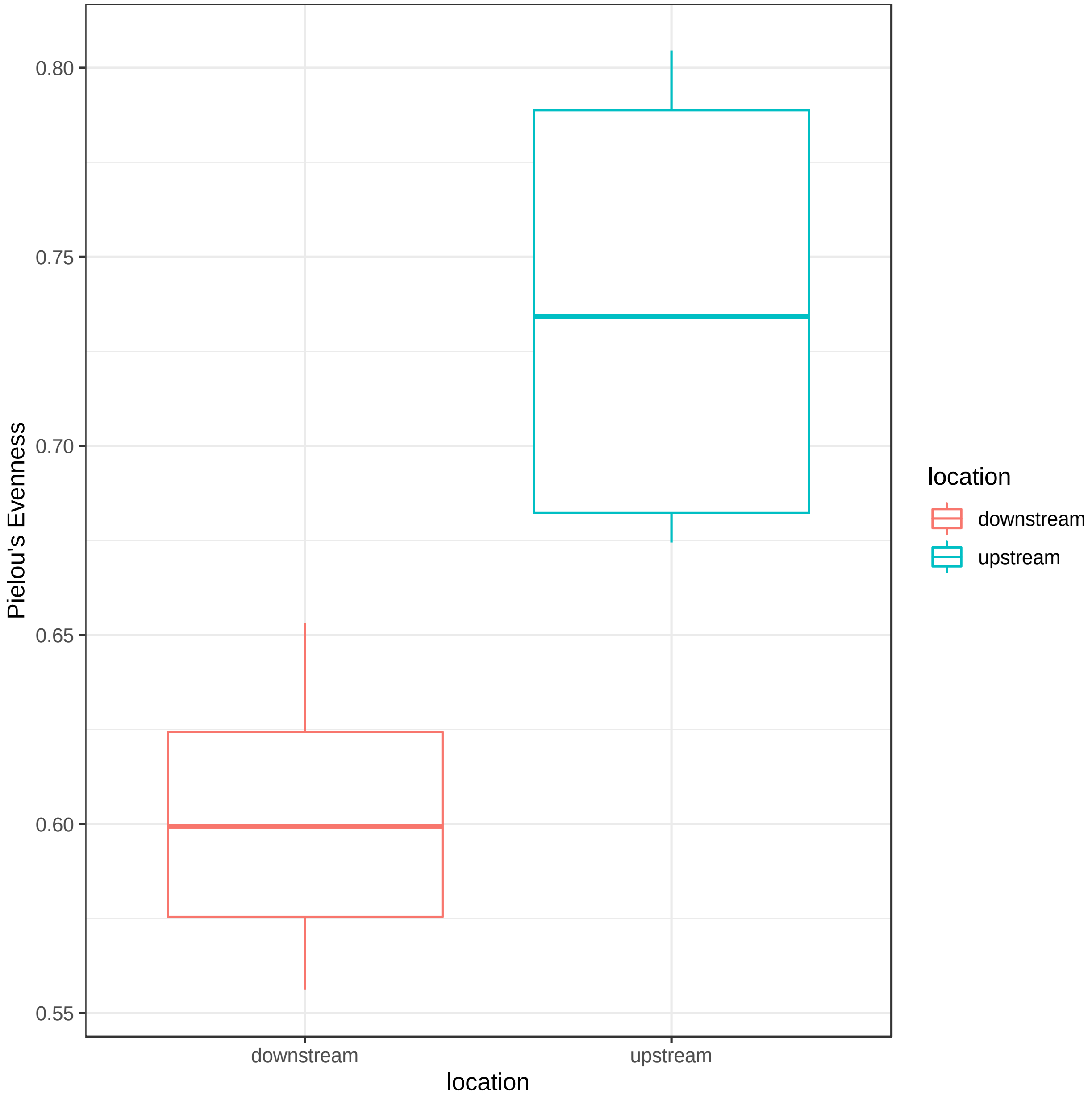

Supplemental Figure 2: Pielou’s evenness, plotted here for hydrocarbon exposed (red) and unexposed (blue) iron mat microbial communities, was significantly different between hydrocarbon exposed and unexposed iron mat microbial communities (ANOVA F = 17.43, p = 0.0031).

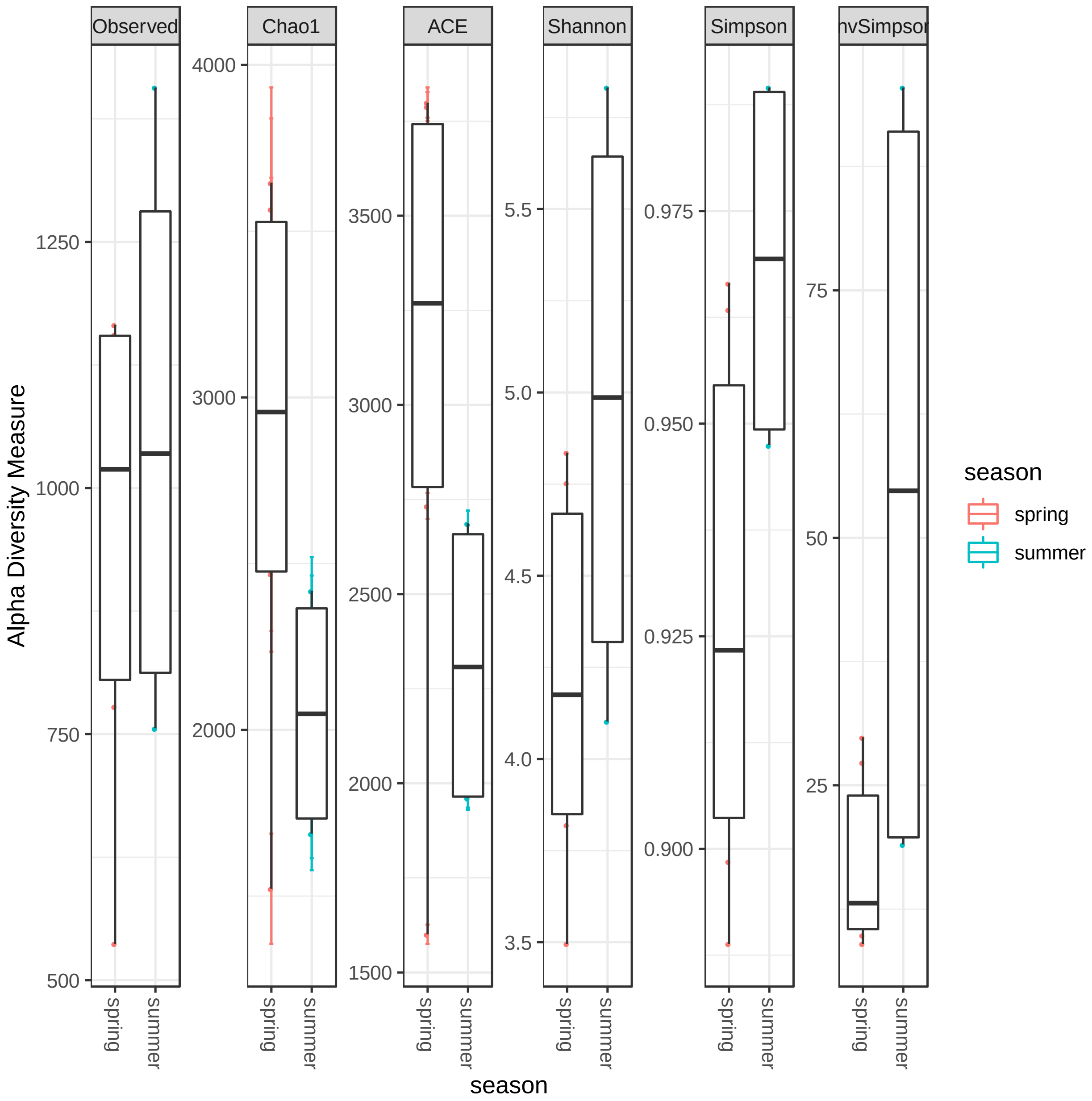

Supplemental Figure 3: Alpha diversity indices compared by season using a Mann-Whitney-Wilcoxon test in R v. 3.5.2. There were no significant differences observed between seasons.

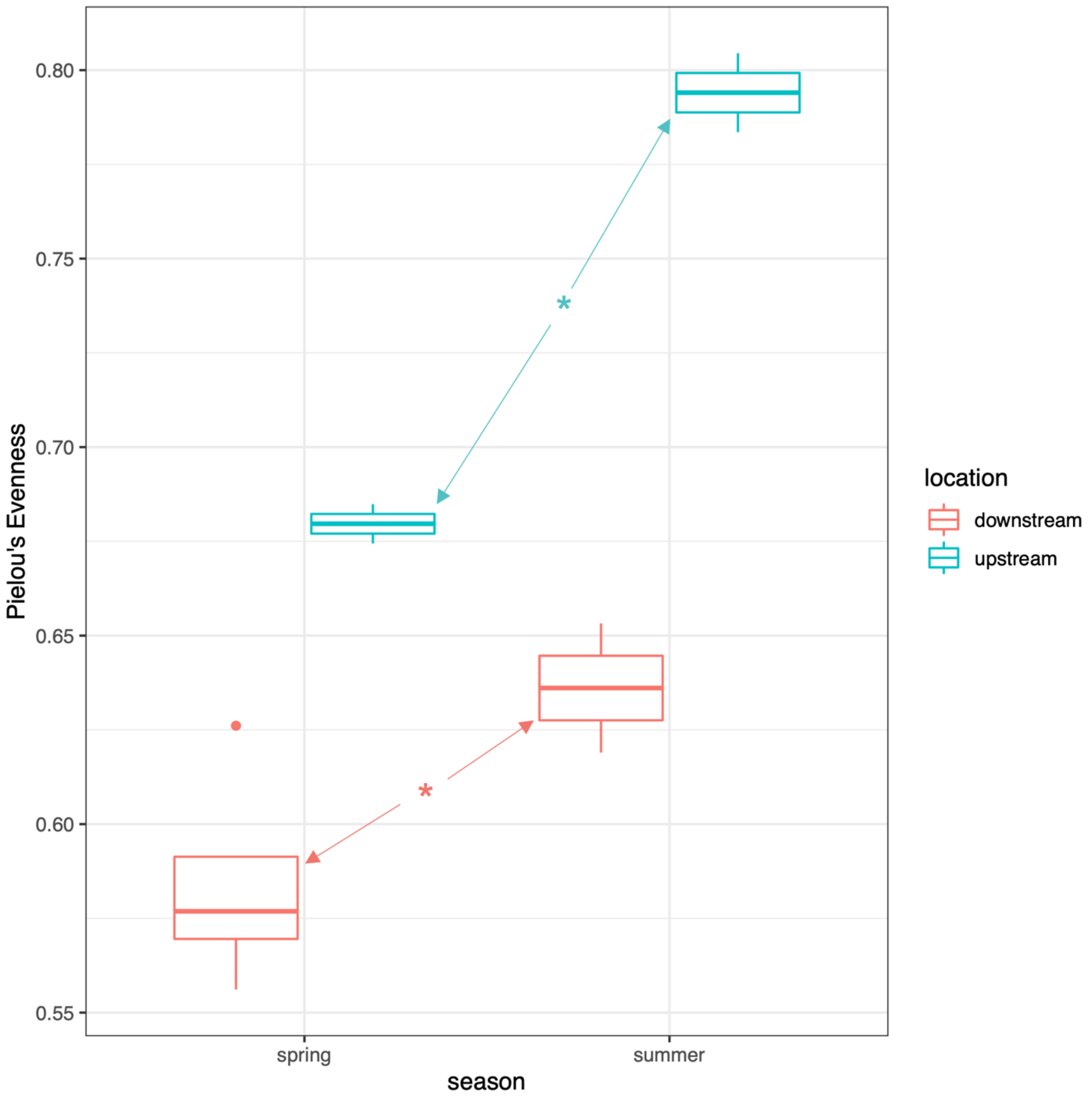

Supplemental Figure 4: Pielou's Evenness compared by season. Pielou's Evenness was not significantly different between spring and summer, however significance was preserved using a model for both season and hydrocarbon exposure (aov(pielou ~ season + location)) (ANOVA season $F = 28.77$, $p = 0.001$, location $F = 42.58$, $p = 0.0003$).

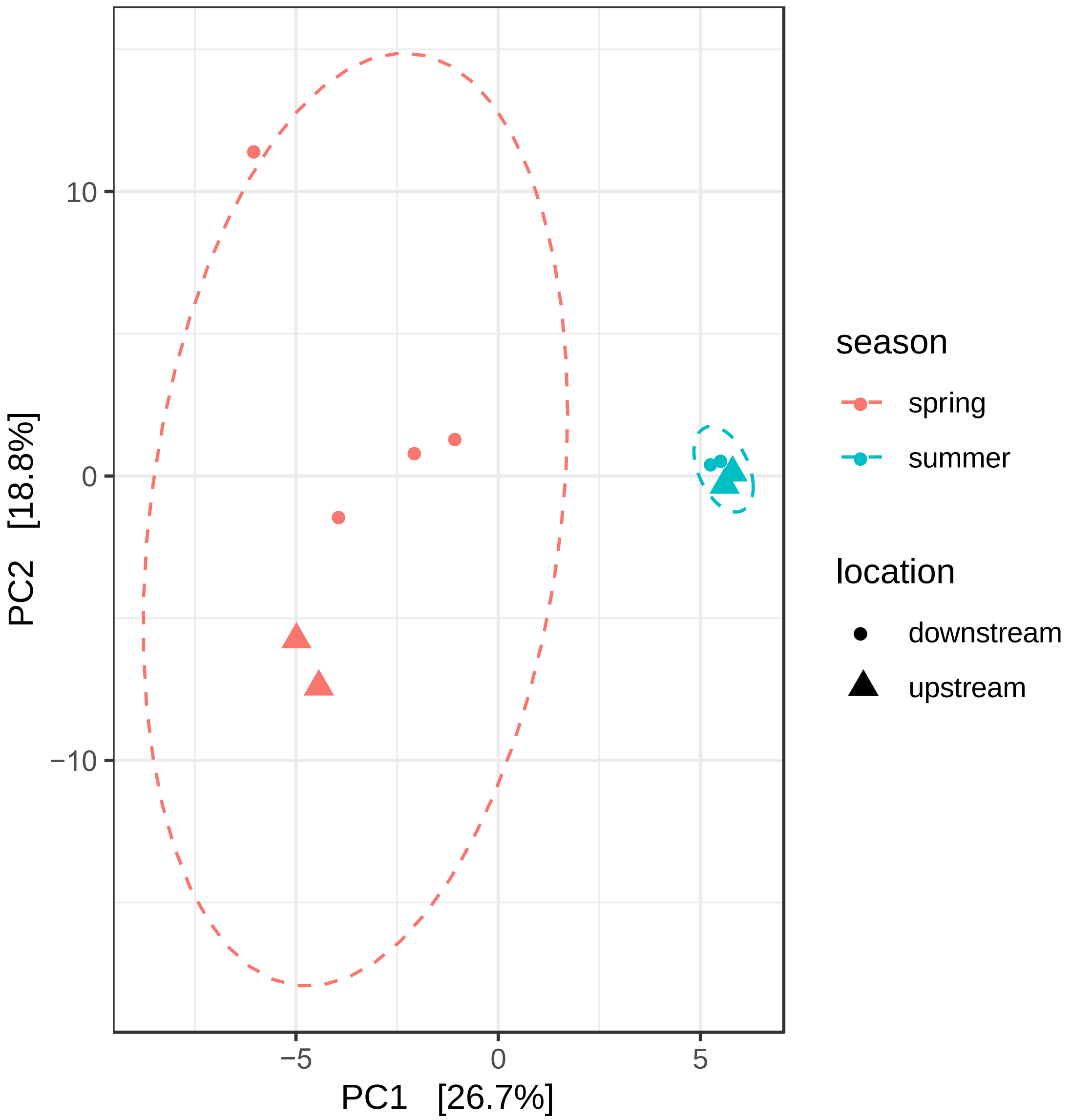

Supplemental Figure 5: Principle components analysis (PCA) of up- and downstream mats by season performed using a redundancy analysis (RDA) of the nine possible axes for the iron mat community data, showing the first two components which explain 26.7% and 18.8% of the total variance explainable by the data collected. Differences between seasons was significant (ADONIS R^2 = 0.25393, p = 0.004, strata = season) suggesting that iron mat communities have a strong signature of species change between seasons.

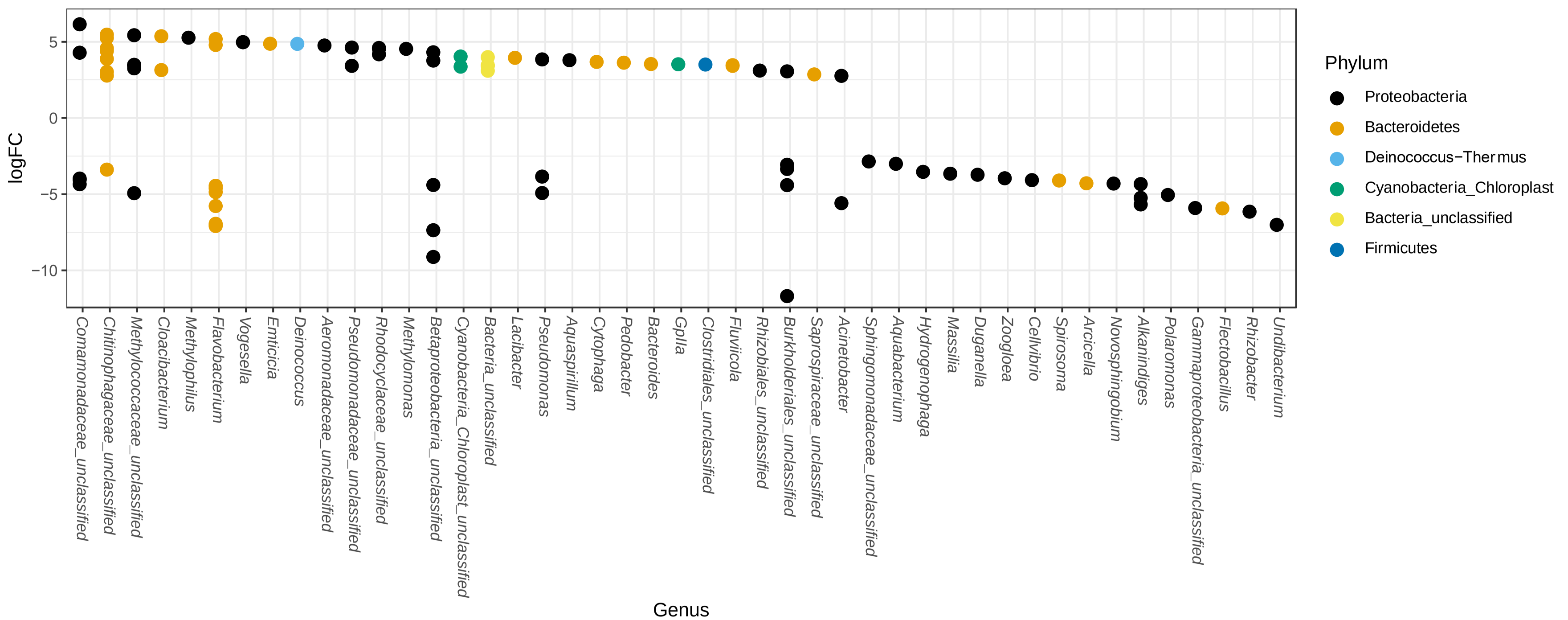

Supplemental Figure 6 : Differential abundances of taxa between iron mats sampled in spring and summer were calculated from an independently filtered data set. Genera with differential abundances with an alpha < 0.001 were plotted. Each point on the plot represents a single OTU sequence. Points with a log Fold Change greater than zero are over-expressed in the summer sampled iron mat communities, whereas OTUs with a log-fold change less than zero are over-expressed in spring communities.

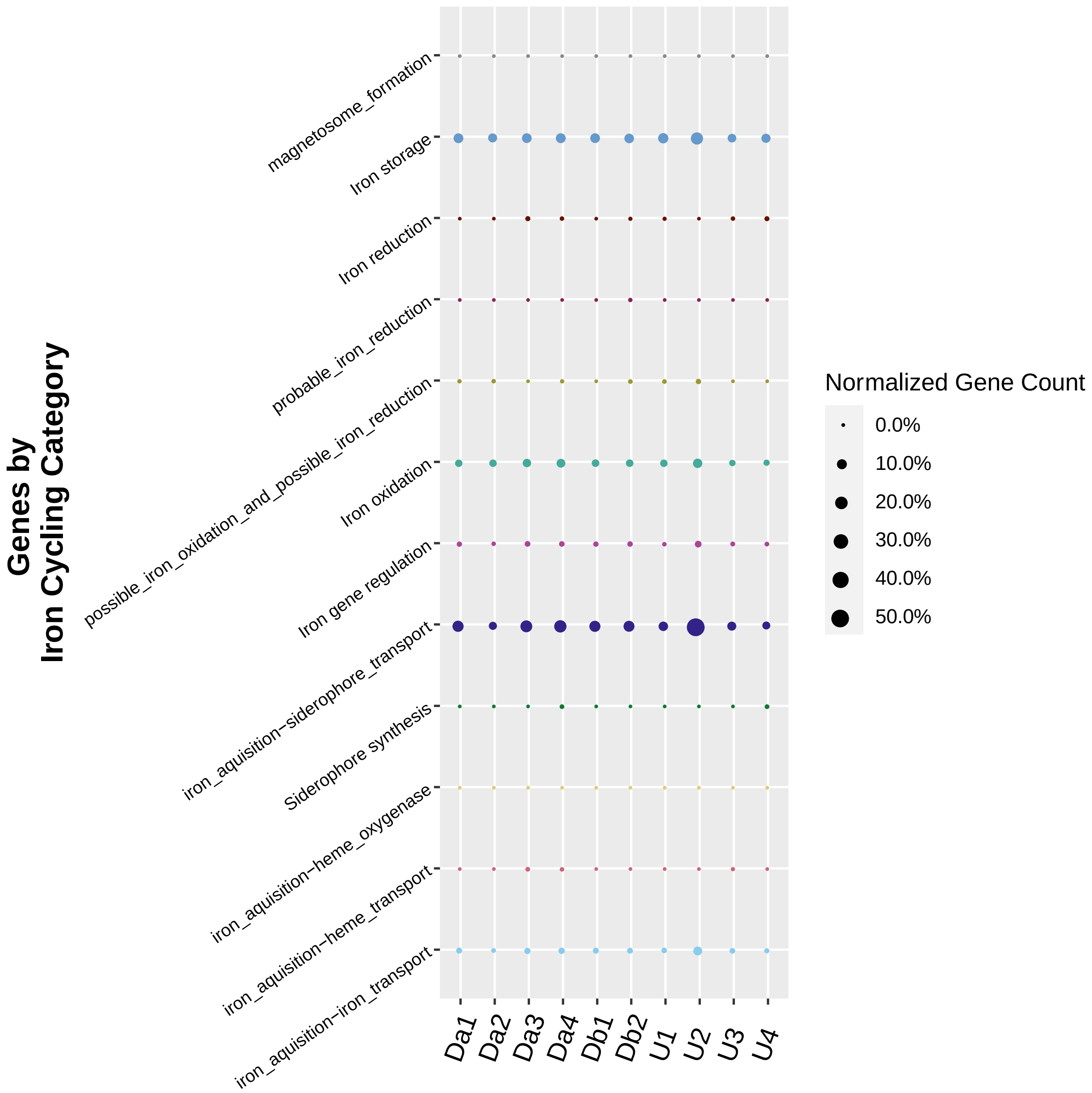

Supplemental Figure 7: Metagenome contig assembly iron-cycling gene count. Metagenome contig assemblies were assessed for iron-cycling genes using FeGenie v. 1 (18). All assemblies returned 0 heme oxygenase or magnetosome sequences, suggesting a paucity of magnetotactic organisms. However, all other iron-cycling gene sequences targeted by the program returned relatively high proportions of total open reading frames (ORFs) in the assemblies. Siderophore transport was the highest at 50% of U2 ORFs.

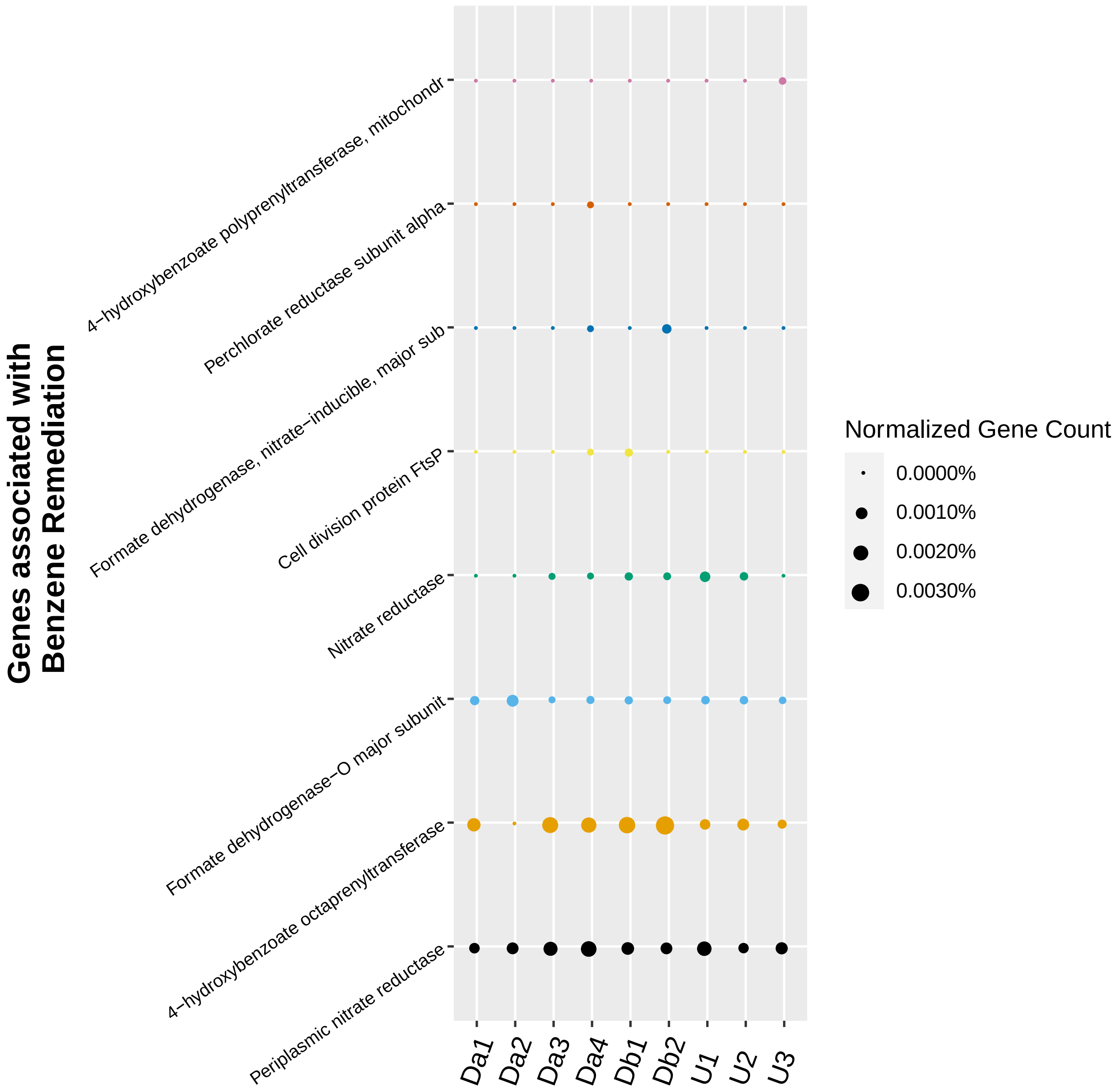

Supplemental Figure 8: Metagenome contig assembly benzene-remediation gene count. Metagenome contig assemblies were assessed for benzene-remediation associated genes using a hidden markov model. Gene counts were normalized for total open reading frames (ORFs). The normalized abundance of benzene-remediation genes was very low, which correlates with the very low relative abundance of 16S rDNA sequences associated with benzene-remediating taxa.

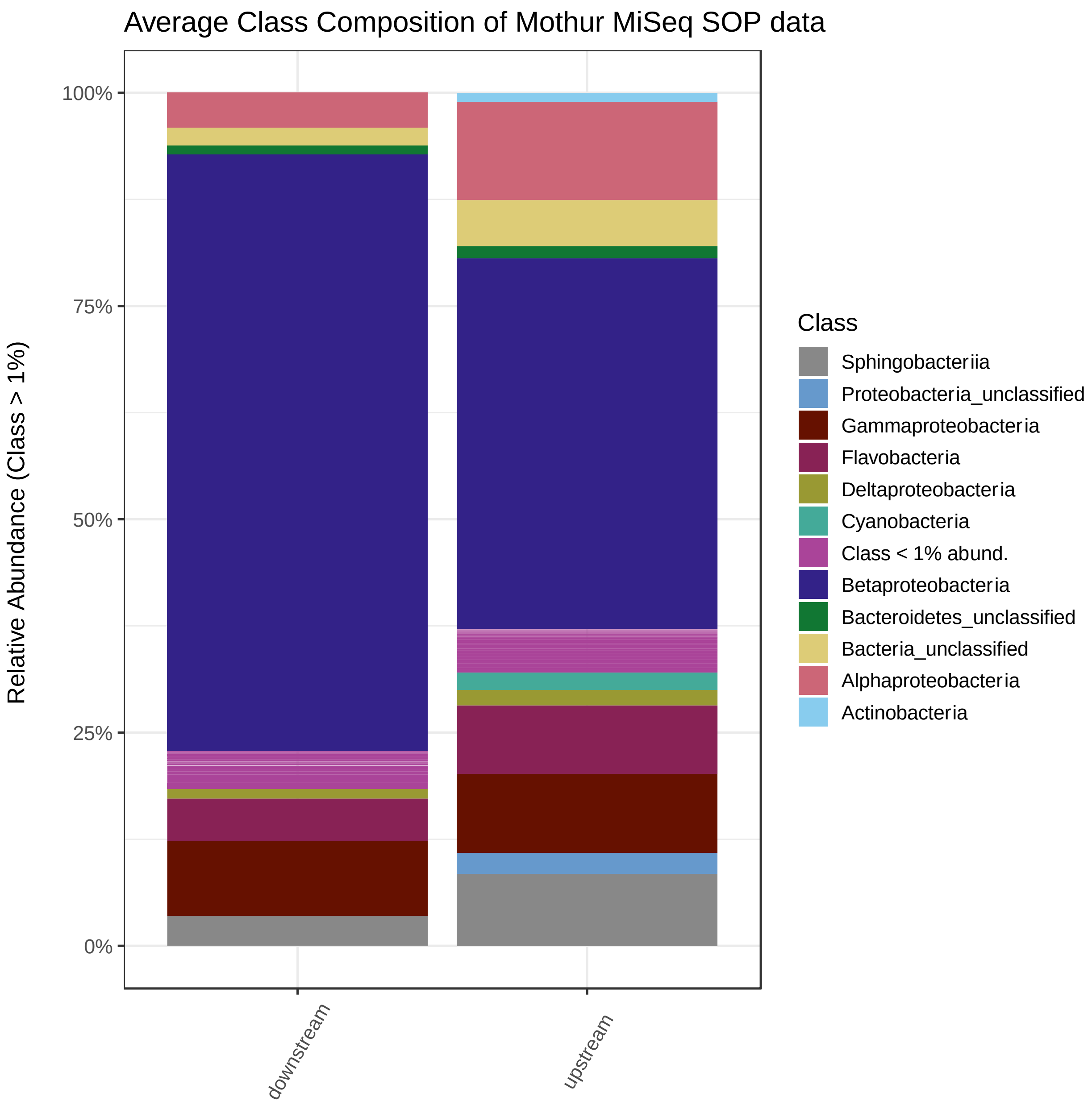

Supplemental Figure 9: Relative abundances of each class calculated and averaged by hydrocarbon exposure. Classes < 1% relative abundance were grouped above under the magenta color. The class Betaproteobacteria had the greatest average relative abundance both up- and downstream from the leaking underground storage tank.

References

1. Kim S-J, Koh D-C, Park S-J, Cha I-T, Park J-W, Na J-H, Roh Y, Ko K-S, Kim K, Rhee S-K. 2012. Molecular analysis of spatial variation of iron-reducing bacteria in riverine alluvial aquifers of the Mankyeong River. The Journal of Microbiology 50:207-217.
2. Coates JD, Ellis DJ, Gaw CV, Lovley DR. 1999. Geothrix fermentans gen. nov., sp. nov., a novel Fe(III)-reducing bacterium from a hydrocarbon-contaminated aquifer. Int J Syst Evol Microbiol 49:1615-1622.
3. Coates JD, Lonergan DJ, Philips EJP, Jenter H, Lovley DR. 1995. *Desulfuromonas palmitatis* sp. nov., a marine dissimilatory Fe(III) reducer that can oxidize long-chain fatty acids. Arch Microbiol 164:406-413.
4. Li X, Lan S-m, Zhu Z-p, Zhang C, Zeng G-m, Liu Y-g, Cao W-c, Song B, Yang H, Wang S-f, Wu S-h. 2018. The bioenergetics mechanisms and applications of sulfate-reducing bacteria in remediation of pollutants in drainage: A review. Ecotoxicol Environ Saf 158:162-170.
5. Leloup J, Fossing H, Kohls K, Holmkvist L, Borowski C, Jørgensen BB. 2009. Sulfate-reducing bacteria in marine sediment (Aarhus Bay, Denmark): abundance and diversity related to geochemical zonation. Environ Microbiol 11:1278-1291.
6. Greene AC. 2014. The Family *Desulfuromonadaceae*, p 143-155. *In* Rosenberg E, DeLong EF, Lory S, Stackebrandt E, Thompson F (ed), The Prokaryotes: Deltaproteobacteria and Epsilonproteobacteria doi:10.1007/978-3-642-39044-9_380. Springer Berlin Heidelberg, Berlin, Heidelberg.
7. Balk M, Altinbaş M, Rijpstra WI, Sinninghe Damsté JS, Stams AJ. 2008. *Desulfatirhabdium butyrativorans* gen. nov., sp. nov., a butyrate-oxidizing, sulfate-reducing bacterium isolated from an anaerobic bioreactor. Int J Syst Evol Microbiol 58:110-5.
8. Rees GN, Patel BK. 2001. *Desulforegula conservatrix* gen. nov., sp. nov., a long-chain fatty acid-oxidizing, sulfate-reducing bacterium isolated from sediments of a freshwater lake. Int J Syst Evol Microbiol 51:1911-1916.
9. Cravo-Laureau C, Labat C, Joulian C, Matheron R, Hirschler-Réa A. 2007. *Desulfatiferula olefinivorans* gen. nov., sp. nov., a long-chain n-alkene-degrading, sulfate-reducing bacterium. Int J Syst Evol Microbiol 57:2699-2702.
10. Suzuki D, Ueki A, Amaishi A, Ueki K. 2007. Desulfopila aestuarii gen. nov., sp. nov., a Gram-negative, rod-like, sulfate-reducing bacterium isolated from an estuarine sediment in Japan. Int J Syst Evol Microbiol 57:520-526.
11. Kuever J. 2014. The Family *Desulfovibrionaceae*, p 107-133. *In* Rosenberg E, DeLong EF, Lory S, Stackebrandt E, Thompson F (ed), The Prokaryotes: Deltaproteobacteria and Epsilonproteobacteria doi:10.1007/978-3-642-39044-9_272. Springer Berlin Heidelberg, Berlin, Heidelberg.

12. Mizuno K, Morishita Y, Ando A, Tsuchiya N, Hirata M, Tanaka K. 2012. Genus-specific and phase-dependent effects of nitrate on a sulfate-reducing bacterial community as revealed by dsrB-based DGGE analyses of wastewater reactors. World Journal of Microbiology and Biotechnology 28:677-686.
13. Parks DH, Imelfort M, Skennerton CT, Hugenholtz P, Tyson GW. 2015. CheckM: assessing the quality of microbial genomes recovered from isolates, single cells, and metagenomes. Genome Res 25:1043-1055.
14. Neely CJ, Graham ED, Tully BJ. 2020. MetaSanity: an integrated microbial genome evaluation and annotation pipeline. Bioinformatics 36:4341-4344.
15. Brettin T, Davis JJ, Disz T, Edwards RA, Gerdes S, Olsen GJ, Olson R, Overbeek R, Parrello B, Pusch GD. 2015. RASTtk: a modular and extensible implementation of the RAST algorithm for building custom annotation pipelines and annotating batches of genomes. Sci Rep 5:8365.
16. Overbeek R, Olson R, Pusch GD, Olsen GJ, Davis JJ, Disz T, Edwards RA, Gerdes S, Parrello B, Shukla M. 2014. The SEED and the Rapid Annotation of microbial genomes using Subsystems Technology (RAST). Nucleic Acids Res 42:D206-D214.
17. Aziz RK, Bartels D, Best AA, DeJongh M, Disz T, Edwards RA, Formsma K, Gerdes S, Glass EM, Kubal M. 2008. The RAST Server: rapid annotations using subsystems technology. BMC Genomics 9:1-15.
18. Garber AI, Nealson KH, Okamoto A, McAllister SM, Chan CS, Barco RA, Merino N. 2020. FeGenie: A Comprehensive Tool for the Identification of Iron Genes and Iron Gene Neighborhoods in Genome and Metagenome Assemblies. Front Microbiol 11.
19. Gurevich A, Saveliev V, Vyahhi N, Tesler G. 2013. QUAST: quality assessment tool for genome assemblies. Bioinformatics 29:1072-1075.
