# Supplementary material for: Microbial community response to hydrocarbon exposure in iron oxide mats: an environmental study
Source: Front Microbiol. 2024 May 10;15:1388973. doi: 10.3389/fmicb.2024.1388973 (PMC11116660; doi:10.3389/fmicb.2024.1388973)
Supplement: Supplementary file 4 [file Data_Sheet_2.PDF]

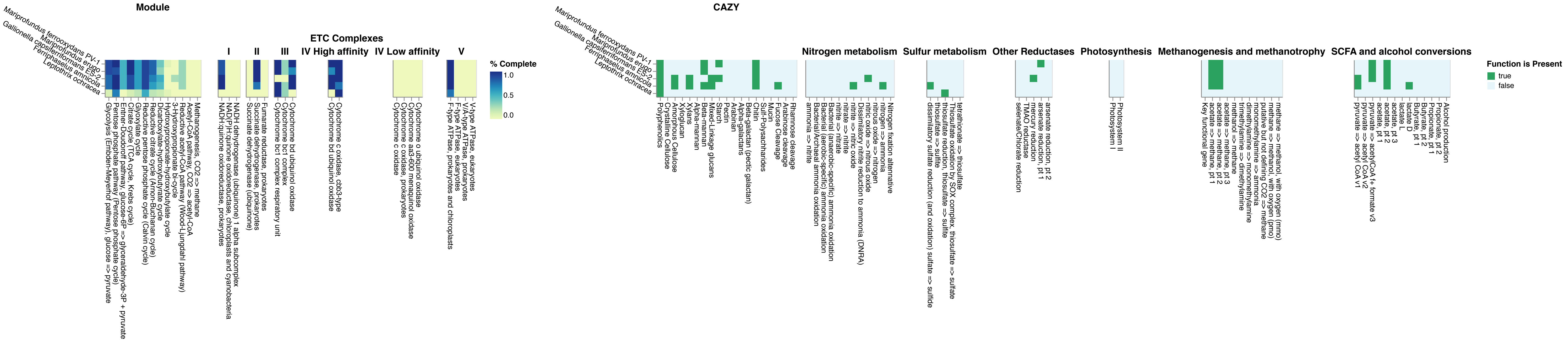

Supplemental File 4: Annotations from DRAM (Distilled and Refined Annotation of Metabolism) of whole genome representative sequences from the NCBI database for the iron-oxidizing bacteria (assembly level in parentheses): *Mariprofundus ferrooxydans* PV-1 (scaffold), *Mariprofundus erugo* (contig), *Gallionella capsiferriformans* ES-2 (complete genome), *Ferriphaselus amnicola* (complete genome), and *Leptothrix ochracea* (scaffold).
