# Supplementary material for: Microbial community response to hydrocarbon exposure in iron oxide mats: an environmental study
Source: Front Microbiol. 2024 May 10;15:1388973. doi: 10.3389/fmicb.2024.1388973 (PMC11116660; doi:10.3389/fmicb.2024.1388973)
Supplement: Supplementary file 5 [file Data_Sheet_3.PDF]

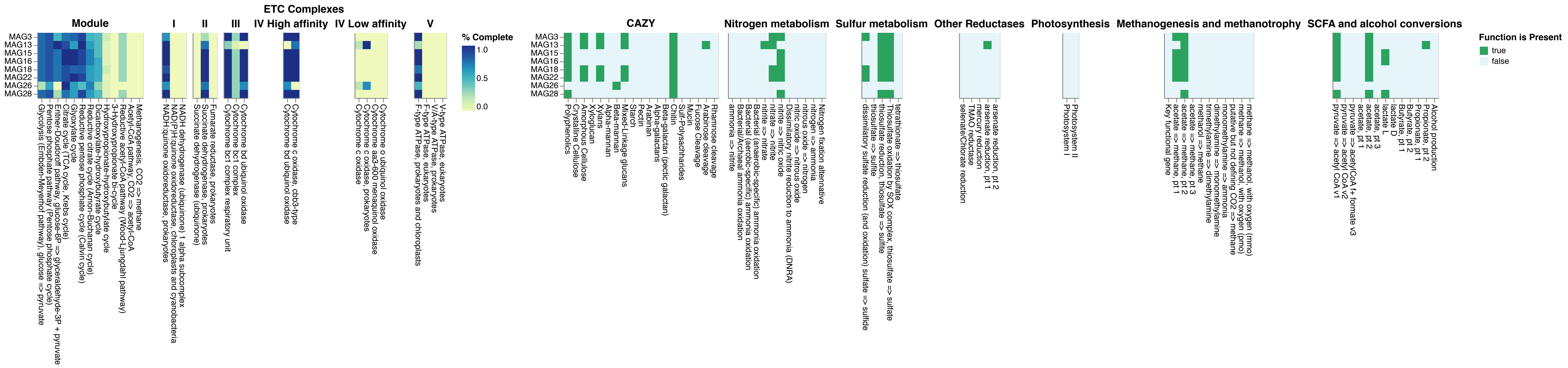

Supplemental File 5: Annotations from DRAM (Distilled and Refined Annotation of Metabolism) of MAGs from this study assigned to the *Burkholderiaceae* family.
